# Accelerated brain aging in patients with major depressive disorder and its neurogenetic basis: evidence from neurotransmitters and gene expression profiles

**DOI:** 10.1017/S0033291725000418

**Published:** 2025-03-05

**Authors:** Haowei Dai, Lijing Niu, Lanxin Peng, Qian Li, Jiayuan Zhang, Keyin Chen, Xingqin Wang, Ruiwang Huang, Tatia M.C. Lee, Ruibin Zhang

**Affiliations:** 1 Laboratory of Cognitive Control and Brain Health, Department of Psychology, School of Public Health, Southern Medical University, Guangzhou, PRC China; 2 Department of Neurosurgery, Institute of Brain Diseases, Nanfang Hospital of Southern Medical University, Guangzhou, PRC China; 3 School of Psychology, South China Normal University, Guangzhou, China.; 4 State Key Laboratory of Brain and Cognitive Sciences, The University of Hong Kong, Hong Kong, SAR China; 5 Laboratory of Neuropsychology and Human Neuroscience, The University of Hong Kong, Hong Kong, SAR China; 6 Guangdong-Hong Kong-Macao Greater Bay Area Center for Brain Science and Brain-Inspired Intelligence, Guangdong-Hong Kong Joint Laboratory for Psychiatric Disorders, Guangdong Basic Research Center of Excellence for Integrated Traditional and Western Medicine for Qingzhi Diseases, Guangzhou; 7 Department of Psychiatry, Zhujiang Hospital, Southern Medical University, Guangzhou, PRC China

**Keywords:** brain aging, cortical thickness, major depressive disorder, transcriptomics methods

## Abstract

**Background:**

Numerous studies have explored the relationship between brain aging and major depressive disorder (MDD) and attempted to explain the phenomenon of faster brain aging in patients with MDD from multiple perspectives. However, a major challenge in this field is elucidating the ontological basis of these changes. Here, we aimed to explore the relationship between brain structural changes in MDD-related brain aging and neurotransmitter expression levels and transcriptomics.

**Methods:**

Imaging data from 670 Japanese participants (MDD: health controls = 233:437) and the support vector regression model were utilized to predict and compare brain age between MDD patients and healthy controls. A map of differences in cortical thickness was generated, furthermore, spatial correlation analysis with neurotransmitters and correlation analysis with gene expression were performed.

**Results:**

The degree of brain aging was found to be significantly higher in patients with MDD. Moreover, significant cortical thinning was observed in the left ventral area, and premotor eye field in patients with MDD. A significant correlation was observed between MDD-related cortical thinning and neurotransmitter receptors/transporters, including dopaminergic, serotonergic, and glutamatergic systems. Enriched Gene Ontology terms, including protein binding, plasma membrane, and protein processing, contribute to MDD-related cortical thinning.

**Conclusions:**

The findings of this study provide further evidence that patients with MDD experience more severe brain aging, deepening our understanding of the underlying neural mechanisms and genetic basis of the brain changes involved. Additionally, these findings hold promise for the development of interventions aimed at preventing further deterioration in MDD-related brain aging, thus offering potential therapeutic avenues.

## Introduction

Brain aging is a complex and multifactorial process. The prevalence of age-related cognitive decline is expected to increase as the global population ages (Dinsdale et al., 2021; Vos et al., 2012). Although the exact mechanisms underlying brain aging remain uncertain, several risk factors have been identified, including genetic factors, lifestyle factors, environmental factors, and clinical pathologies such as mental disorders (Elliott et al., 2021; Kuo et al., 2020). Previous studies have suggested that major depressive disorder (MDD) may be a potential contributor to brain aging (Kaufmann et al., 2019; Koutsouleris et al., 2014), which might, in turn, contribute to neurodegenerative conditions such as Alzheimer’s disease (AD). For example, an increasing number of studies indicate a close link between MDD and AD (Babcock, Page, Fallon, & Webb, 2021). Specifically, patients with MDD exhibit significantly higher incidence rates of AD (Huang, Weng, Wang, & Hsieh, 2021). As such, it is worth exploring whether MDD contributes to brain aging. Therefore, further research is necessary to clarify the relationship between MDD and brain aging and to identify potential interventions that could help to prevent or mitigate the effects of brain aging.

MDD, a mental disorder characterized by persistent feelings of sadness and a loss of interest in activities (Marx et al., 2023), has been observed to impact brain function, potentially accelerating the process of brain aging (Penninx, Milaneschi, Lamers, & Vogelzangs, 2013). This process is attributed to alterations in neurobiological mechanisms within the brain, such as neuroplasticity and neuroinflammatory responses, due to MDD (Fries, Saldana, Finnstein, & Rein, 2023). This association emphasizes the importance of identifying patterns of brain aging in individuals with MDD to ascertain whether, and how, they deviate from healthy aging trajectories. Age-related changes in cortical thickness, surface area, and subcortical volumes vary in patients with MDD, which potentially indicates that the morphology of the brain might be influenced by various developmental pathways and that individuals of the same chronological age may have different brain ages (Cole et al., 2018; Kaufmann et al., 2019; Leonardsen et al., 2022). A metric that quantifies the difference between chronological age and brain age is therefore essential, with neuroimaging being the preferred method for obtaining biomarkers of brain aging, as the existing diverse neuroimaging modalities can reliably and accurately measure detailed structural and functional information about various biological characteristics of the brain. In this context, brain-predicted age deviation (brain-PAD), derived from predicted brain age based on neuroimaging data (Dörfel et al., 2023; Liem et al., 2017), has significant potential in identifying individuals at risk of age-related disease and detecting potential clinical pathologies (Elliott et al., 2021). Research has shown that individuals with MDD may have a significantly higher brain-PAD compared to healthy controls (Han et al., 2021; Luo, Chen, Qiu, & Jia, 2022). This further supports the notion that MDD may be a potential clinical pathology that contributes to brain aging.

Recent studies have explored the relationship between MDD and brain aging, using machine learning methods to process structural magnetic resonance imaging (sMRI) and functional magnetic resonance imaging (fMRI) data to predict brain age in individuals of Caucasian descent. In these studies, the MDD group showed a significantly higher brain-PAD than healthy controls (Christman et al., 2020; Han et al., 2021; Kaufmann et al., 2019; Koutsouleris et al., 2014). However, some studies have suggested that MDD-related brain changes can be explained by underlying genetic mechanisms involving brain development and plasticity (Han et al., 2021; Leonardsen et al., 2023). More importantly, variability in gene expression exists among diverse populations of various nationalities and ethnicities. For example, a comprehensive genome-wide association study (GWAS) has shown a remarkably small overlap between risk loci associated with MDD between individuals of East Asian and Caucasian descent (Giannakopoulou et al., 2021). Hence, it is imperative to conduct studies on brain aging in patients with MDD of other ethnicities (i.e. East Asian populations).

Beyond the urgency to identify brain aging patterns in other ethnicities, it is crucial to bridge the gap in understanding the molecular and cellular mechanisms underlying brain aging. The main limitation of previous studies is their reliance on the analysis of imaging-derived phenotypes (IDPs) (Han et al., 2021; Luo et al., 2022; Qiu et al., 2023), such as functional connectivity and cortical thickness, derived solely from MRI scans of patients with MDD and healthy controls. Imaging-derived phenotypes provide indirect measures of pathological mechanisms but lack specificity about the underlying molecular and cellular characteristics of brain tissue (Cassidy & Radda, 2005). The analysis of imaging transcriptomics data allows us to combine molecular tissue information with IDPs, thus offering a novel perspective on how spatial patterns of gene expression relate to anatomical changes in brain structure and function in patients with MDD (Fornito, Arnatkevičiūtė, & Fulcher, 2019). In recent years, the combination of brain imaging and other techniques for the analysis of physiological data (Arnatkeviciute, Markello, Fulcher, Misic, & Fornito, 2023) has provided new methods for investigating the structural and functional changes associated with MDD from both neurochemical and biochemical perspectives. Therefore, it is critical to combine transcriptome-connectome analysis to investigate the relationship between structural changes associated with MDD that may lead to brain aging and gene expression profiles characteristic of this disease. Furthermore, to provide a comprehensive understanding of MDD and better elucidate clinical symptoms, it is necessary to establish a link between the observed MDD-related alterations in brain structures and neurotransmitter systems. Investigating neurotransmitter expression levels is imperative for understanding the molecular basis of the psychopathological processes underlying MDD. Prior studies have indicated a close association between the expression levels of certain neurotransmitters, such as serotonin 5-hydroxytryptamine receptor and dopamine, and the onset and progression of MDD (Fries et al., 2023; Mallet, Gorwood, Le Strat, & Dubertret, 2019). However, further investigation is needed to explicitly determine whether this relationship is linked to brain aging. The multimodal data analysis methods used in our study will enhance our understanding of the potential neural mechanisms and biological and molecular genetic bases underlying the structural changes associated with MDD that contribute to brain aging, providing a foundation for future intervention interventions.

Herein, our study aims to overcome the limitations of previous research, which primarily relied on imaging-derived phenotypes (IDPs) without integrating molecular and cellular-level information, by utilizing validated sMRI features and a support vector regression (SVR) model within the ENIGMA framework (Han, et al., 2021) to predict brain age in East Asian populations. Furthermore, this study bridges the gap by examining MDD-related structural changes, such as cortical thickness alterations, and linking these changes to neurotransmitter expression levels and transcriptomic profiles, providing a more comprehensive understanding of brain aging mechanisms relative to healthy controls. We hypothesize that (1) the brain-PAD of patients with MDD is significantly higher than that of the healthy controls; (2) cortical thickness in certain brain regions is significantly lower in patients with MDD; and (3) cortical thickness changes in patients with MDD are associated with expression levels of neurotransmitters and enriched Gene Ontology (GO) terms.

## Methods

### Overview

**Figure 1.**
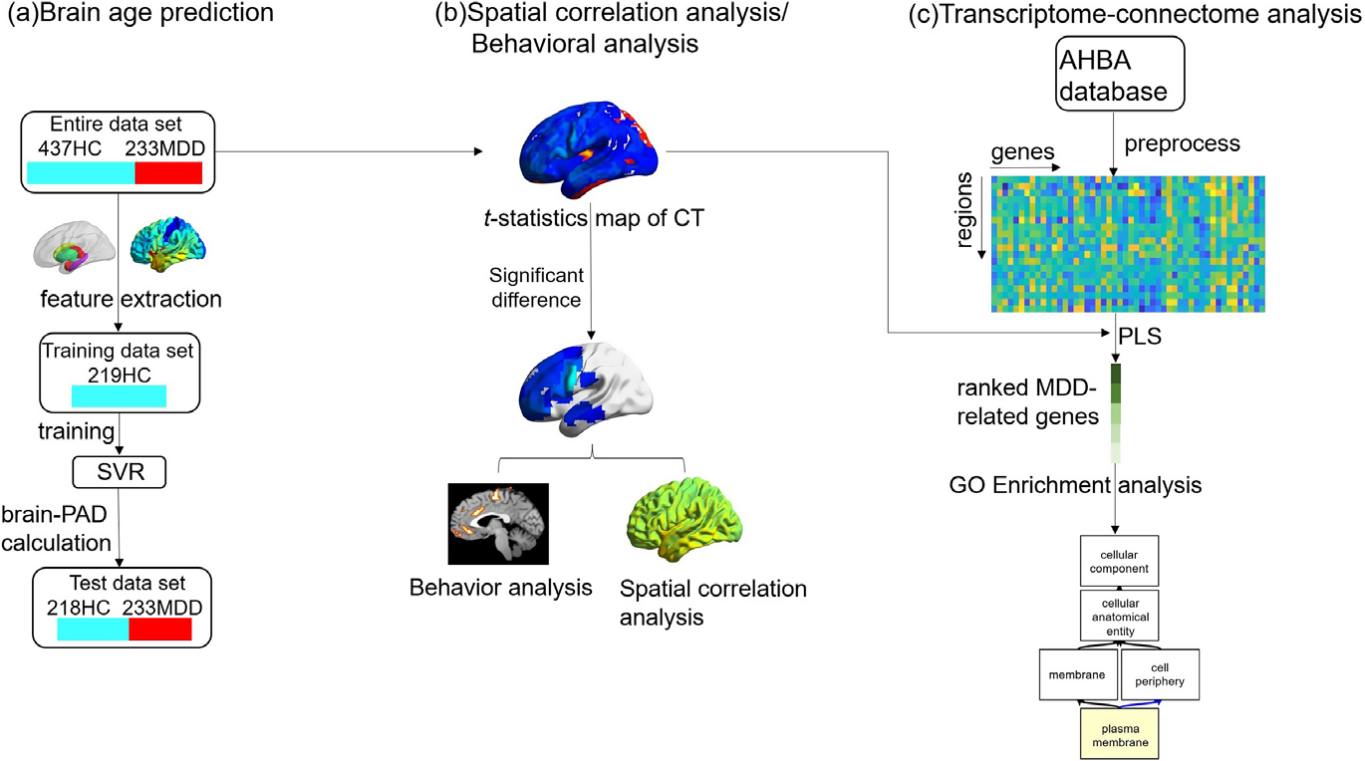
Overview of the analysis pipeline. (a) Brian age prediction: structural MRI data was preprocessed and cortical thickness and subcortical volume features were extracted. Half of the healthy participants were randomly selected as the training dataset to train the support vector regression (SVR) model and the rest participants consisted of the test dataset to evaluate the performance of the trained SVR model. (b) Spatial correlation analysis/behavioral analysis: A *t*-statistics map was computed to identify regions of significant cortical thickness differences between the MDD and HC groups. The behavior analysis plugin in Mango was used to compare the *t*-map obtained from brain regions exhibiting significant differences in cortical thickness between individuals with MDD and healthy controls with the behavioral domains associated with cognitive terms to decode the related behavioral processes. Meanwhile, the spatial relationship between the *t*-map of cortical thickness (CT) difference and the distribution of receptors/transporters was calculated. (c)Transcriptome-connectome analysis: gene expression profiles from the Allen Human Brain Atlas (AHBA) across six postmortem brains. The relationship between changes in cortical thickness associated with MDD and gene expression from the AHBA was investigated and gene ontology enrichment analysis on the MDD-relevant genes was performed.

The analytical framework is summarized in Figure 1. First, we divided the participants into training and testing sets to train and test the SVR model (Figure 1a). Next, we constructed a *t*-statistics map to determine which regions of interest (ROIs) that showed significant differences in cortical thickness between the MDD and healthy control (HC) groups. The significant *t*-map was then compared with behavioral domains to decode the associated behavioral processes. The spatial relationship between the significant *t*-map and the distribution of receptors/transporters was calculated to investigate the association between the altered cortical thickness in MDD and the expression of neurotransmitter receptors/transporters (Figure 1b). Finally, the microarray-based gene expression data and *t*-map were included in a partial least squares (PLS) regression analysis to examine the relationship between transcriptome profiles and cortical thickness changes associated with MDD-related brain aging. GO enrichment analysis was then performed to identify enriched GO terms (Figure 1c).

### Samples

Patients with MDD and HCs were selected from the multi-disorder MRI dataset in a Japanese public database that includes 5 sites (Tanaka et al., 2021), including 239 MDD patients and 439 healthy controls (https://doi.org/10.7303/syn22317079). More detailed information for each site is shown in Supplementary Table 1. Our focus was solely on the analysis of sMRI data. More detailed information on the imaging parameters for T1-weighted (T1w) sMRI is provided in Supplementary Table 2. Following a rigorous examination of image quality, eight participants (including six MDD patients and two healthy controls) were found to have unreadable T1 images. Consequently, these individuals were excluded from subsequent analyses. The remaining 670 subjects were included in the further analysis. There were no significant differences in chronological age between the patients with MDD and HCs (*t* = 1.117, *p* = 0.264).

### Data preprocessing and feature selection

Cortical thickness was calculated using CAT12 (http://dbm.neuro.uni-jena.de/cat), an extension of the SPM12. Although the T1 images were segmented into gray matter, white matter, and cerebrospinal fluid, we only extracted the cortical thickness and subcortical volume. The extracted cortical thickness was resampled to a higher resolution 164 k mesh compatible with Freesurfer data and, finally, resampled cortical thickness was smoothed.

We constructed a 670 × 372 feature matrix including cortical thickness and subcortical volumes. Cortical parcellations were based on the Human Connectome Project (HCP) atlas (Glasser et al., 2016), which utilizes a semi-automated neuroanatomical approach combined with machine learning classifiers, enabling precise identification and parcellation of cortical regions at the individual level. Subcortical parcellations were derived form the Harvard-Oxford Structural Atlas (HOA). We utilized Gretna (Wang et al., 2015) to obtain cortical thickness measurements for 358 regions of interest (ROIs) (the hippocampus was excluded because of its low count in vertices in both hemispheres) and subcortical volume measurements for 14 ROIs.

### Model training and evaluation

Machine learning is particularly accurate in predicting brain age because it is well-suited for analyzing high-dimensional datasets such as those commonly derived from neuroimaging studies that typically include measurements from hundreds of thousands of voxels. Over the past decade, a growing number of studies have used neuroimaging data to predict age, with most studies employing voxel-based gray matter or cortical thickness as features (Gutierrez Becker et al., 2018; Valizadeh, Hänggi, Mérillat, & Jäncke, 2017). All of these studies have confirmed the reliability of using machine learning algorithms and T1-weighted MRI for brain age prediction. In machine learning, Linear SVR is a supervised learning technique based on the priciples of Support Vector Machines. It is commonly employed for predicting continuous variables such as age. It is worth noting that SVR has been successfully applied in previous studies assessing patterns of brain aging (Dosenbach et al., 2010; Liem et al., 2017). In this research, we employed the SVR model to train a brain age prediction model. The training samples comprised 219 randomly selected HCs, while the remaining 233 individuals with MDD and 218 HCs formed the test samples for subsequent age prediction and between-group comparison of brain-PAD. To enhance the statistical power of the trained model and mitigate the impact of random sampling errors, we randomly shuffled the labels of the training samples 10,000 times. Subsequently, for each permutation, the model was retrained to construct a distribution of brain-PAD differences, which were then compared to the brain-PAD differences from the initially trained model.

The performance of the model was further validated in the test dataset. The trained model was applied to the patients with MDD and HCs in the test set to obtain brain-based age estimates. To assess the model’s performance, we computed the Pearson correlation coefficient between the predicted and chronological ages. Finally, we employed a two-sample *t*-test to compare the brain-PAD between patients with MDD and healthy controls in the test dataset, aiming to determine whether our trained model could effectively distinguish the difference in brain aging between patients with MDD and HCs.

### Creation of cortical thickness difference map by harmonization

The SVR model only yields information on the relative importance of each feature, without explicitly determining the significant contributions of the cortical thickness of specific brain regions to brain aging. Previous research has indicated that brain regions associated with depression tend to make a greater contribution to brain aging (Luo et al., 2022). Therefore, we speculate that brain regions with significant changes in cortical thickness in individuals with MDD may also play a more substantial role in brain aging. Therefore, the mechanisms underlying MDD-related brain aging can be explored by investigating the brain regions that exhibit significant differences (e.g. via a *t*-statistics map) in cortical thickness between patients with MDD and HCs. To obtain a reliable map showing differences in cortical thickness between patients with MDD and HCs, we used Combat (https://github.com/Jfortin1/ComBatHarmonization) to harmonize individual cortical thickness maps from five sites (Fortin et al., 2017), controlling for the effects of age and sex. After harmonization, we performed a two-sample *t*-test to compare ROI-based differences in cortical thickness between patients with MDD and HCs. Parcellations were based on the HCP atlas. The false discovery rate (FDR) method was used to correct multiple comparisons with a significance threshold of *q* < 0.05. Then, a correlation analysis was conducted between weights of cortical thickness features in the prediction model and the *t*-map. Finally, we identified the ROIs with significant *t*-statistic and calculated Pearson correlation coefficients between the cortical thickness of these regions and predicted brain age, controlling for age, site, and sex.

### Analysis of the association between behavioral domains and MDD-related alterations in cortical thickness

To further explore the association between MDD-related cortical thickness alterations and specific cognitive processes (Poldrack, 2006), aiming to elucidate the functional relevance of brain regions, we used the behavioral analysis plugins available in Mango (Lancaster et al., 2012). We compared the *t*-map for the differences in cortical thickness between patients with MDD and HCs with behavioral domains related to cognitive terms available in BrainMap. Our objective was to decipher the behavioral processes or cognitive terms associated with cortical thickness alterations related to MDD and validate whether the identified brain regions, obtained by computing the differences in cortical thickness between patients with MDD and HCs, are indeed implicated in MDD. The results of the behavior analysis are presented for the five behavioral domains and sixty sub-domains of BrainMap.

### Spatial correlation between MDD-related alterations in cortical thickness and neurotransmitter receptors/transporters

To investigate the relationship between altered cortical thickness in MDD and the expression of specific neurotransmitter receptors/transporters, we computed the spatial relationship between MDD-related cortical thickness alterations and the distribution of these receptors/transporters using the JuSpace toolbox (Dukart et al., 2021). We computed the Spearman correlation between changes in cortical thickness and the distribution of relevant receptor/transporter profiles, and performed a Fisher’s z transformation for these coefficients to gain insights into the neurobiological mechanisms underlying MDD (Supplementary Method 1). We investigated positron emission tomography (PET) or single-photon emission computed tomography (SPECT) maps, including those for the expression level of serotonin 5-hydroxytryptamine receptor subtype 1a (5-HT1a), 5-HT subtype 1b (5-HT1b), 5-HT subtype 2a (5-HT2a), dopamine D1 (D1), dopamine D2 (D2), dopamine transporter (DAT), dopamine synthesis capacity (F-DOPA), gamma-aminobutyric acid (GABAa), and glutamatergic receptor (mGluR5).

### Analysis of the association between gene expression and cortical thickness alterations in patients with MDD

Microarray-based gene expression data from the Allen Human Brain Atlas was preprocessed following a previously reported pipeline (Arnatkevic̆iūtė, Fulcher, & Fornito, 2019) (detailed information is provided in Supplementary Method 2). We performed a partial least squares (PLS) regression analysis to explore the association between gene expression and the *t*-map of between-group differences in cortical thickness (Abdi, 2010). The gene expression data and the *t*-map of between-group cortical thickness differences were used as the predictor and response variables, respectively. A spatial autocorrelation corrected permutation test (number of permutations: 10000) was conducted by shuffling the *t*-map to check whether the 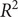 of the PLS component was significantly greater than that expected by chance. For the significant component, the bootstrapping method was employed for gene expression data and the cortical thickness *t*-map to correct the estimation error of the weight of each gene (Whitaker et al., 2016). The genes were ranked in descending order based on the corrected weights, representing their contribution to the PLS regression component.

To determine whether MDD-related genes contributed to the PLS model, we defined MDD risk genes based on a GWAS study including 46 MDD risk loci and mapped these 46 MDD risk loci to 147 genes (Giannakopoulou et al., 2021; Schizophrenia Working Group of the Psychiatric Genomics Consortium, 2014) (Supplementary Method 3). Only 60 MDD-associated risk genes with qualified AHBA expression data were included in this study and further analysis. We ranked these MDD-related genes in descending order of their corrected weight in significant PLS components. MDD-related genes were included in the gene enrichment analysis to identify enriched GO terms by using KOBAS (Bu et al., 2021). We sought to explore the regions associated with changes in cortical thickness related to brain aging in individuals with MDD and investigated the GO annotations of the MDD gene set that are associated with these cortical thickness changes. Furthermore, we generated directed acyclic graphs illustrating the hierarchical relationships between GO terms. GO terms including biological process, molecular function, and cellular component were considered (Supplementary Method 4). Significant enrichment was set at Benjamini-Hochberg FDR-corrected *q* < 0.05 (Whitaker et al., 2016).

## Validation

### Assessing the replicability of prediction methods through the a2009s Atlas and Combat

To ensure the reproducibility of the results, we further replaced the cortical thickness atlas with the a2009s Atlas (Destrieux et al., 2010). Subsequently, we reconstructed the feature matrix and trained the SVR model. Then, we performed age prediction and between-group comparisons using this updated model to validate the consistency of our findings (Supplementary Result 1). We also performed prediction and between-group comparisons using the feature matrix, applying Combat, and we found the results to be consistent with previous results obtained without Combat (Supplementary Result 4).

### Evaluating the effectiveness of t-map construction

To verify the effectiveness of the difference in cortical thickness calculated by Combat methods, we used another method, namely, regression diagnosis, to construct a *t*-statistic map, controlling for the effects of age, sex, and site, with FDR correction. Furthermore, we calculated the Pearson correlation coefficients between the *t*-statistic values for differences in cortical thickness calculated by the two methods. We also conducted an association analysis between behavioral domains and a *t*-map constructed by regression diagnosis, as well as a spatial correlation analysis between the *t*-map and neurotransmitter receptors/transporters (Supplementary Results 2, 3, Supplementary Table 6, 7).

## Results

### Brain age prediction model performance

**Figure 2.**
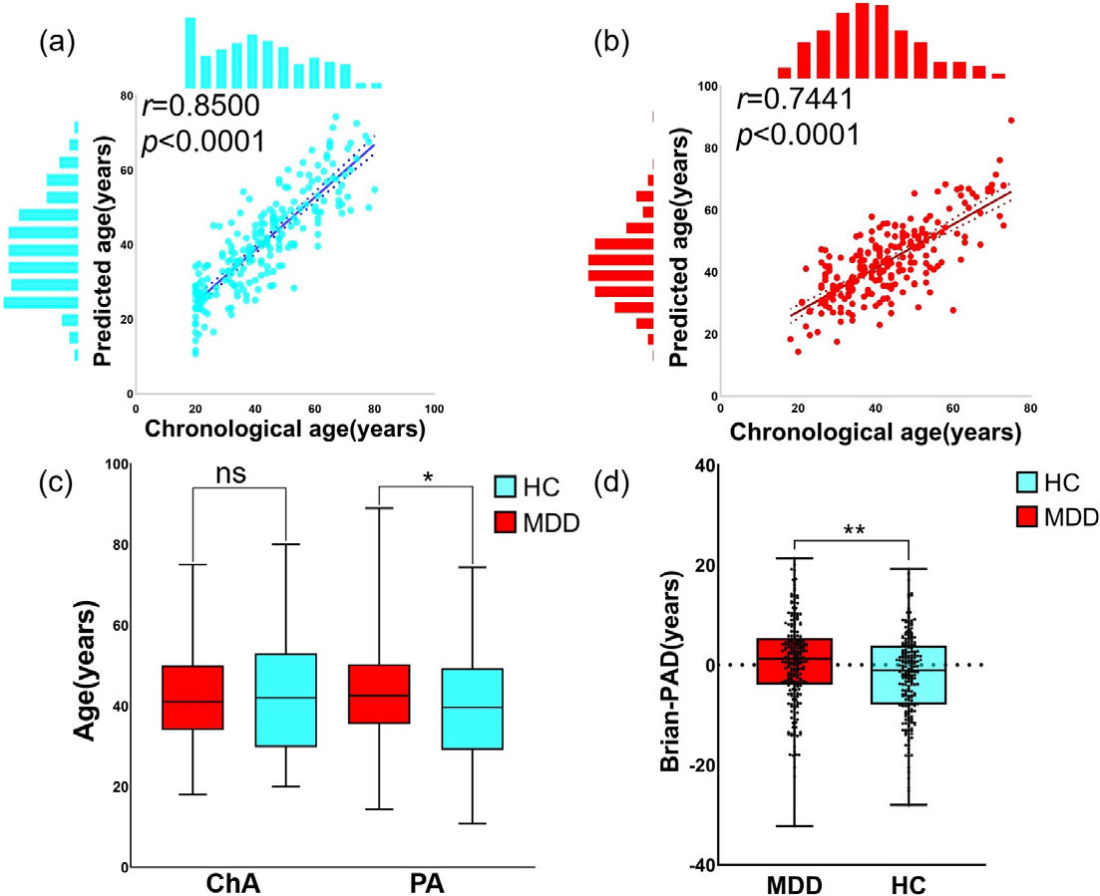
Performance of brain age prediction model. (a) Positive correlation between the chronological age and predicted age in the test healthy controls (HC). (b) Positive correlation between the chronological age and predicted age in the test MDD patients. (c) Group comparison between MDD patients and healthy controls in the test dataset and there is no significant difference in chronological age (ChA) between the two groups, while the predicted age (PA) in MDD patients is significantly higher than that in healthy controls. (d) Group comparison of the brain-PAD between MDD patients and healthy controls in the test dataset and brain-PAD in MDD patients is significantly higher than that in healthy controls. Ns means that the difference between MDD and HC is not significant. *, *p* < 0.05. **, *p* < 0.01.

The model obtained from the training sample was used to estimate the brain age of patients with MDD and the rest of the HCs in the test sample. We found a high positive correlation between chronological age and predicted age in both MDD patients and HCs in the test sample (HC: *r* = 0.8500, *p* < 0.0001; MDD: *r* = 0.7441, *p* < 0.0001 Figure 2a,b). The difference in brain-PAD between HCs and MDD patients in the test sample was found to be significant (Cohen’s d = 0.3014, 95% CI: 0.12–0.49, *p* = 0.0015, Figure 2d). The permutation test also revealed that differences in brain-PAD were significant (*p* = 0.0050). Compared with HCs in the test sample, patients with MDD showed significantly higher predicted age (predicted age: Cohen’s d = 0.2345, 95% CI: 0.05–0.42, *p* = 0.0130, Figure 2c), with no difference in chronological age (*p* = 0.7893).

### MDD-related alterations in cortical thickness

**Figure 3.**
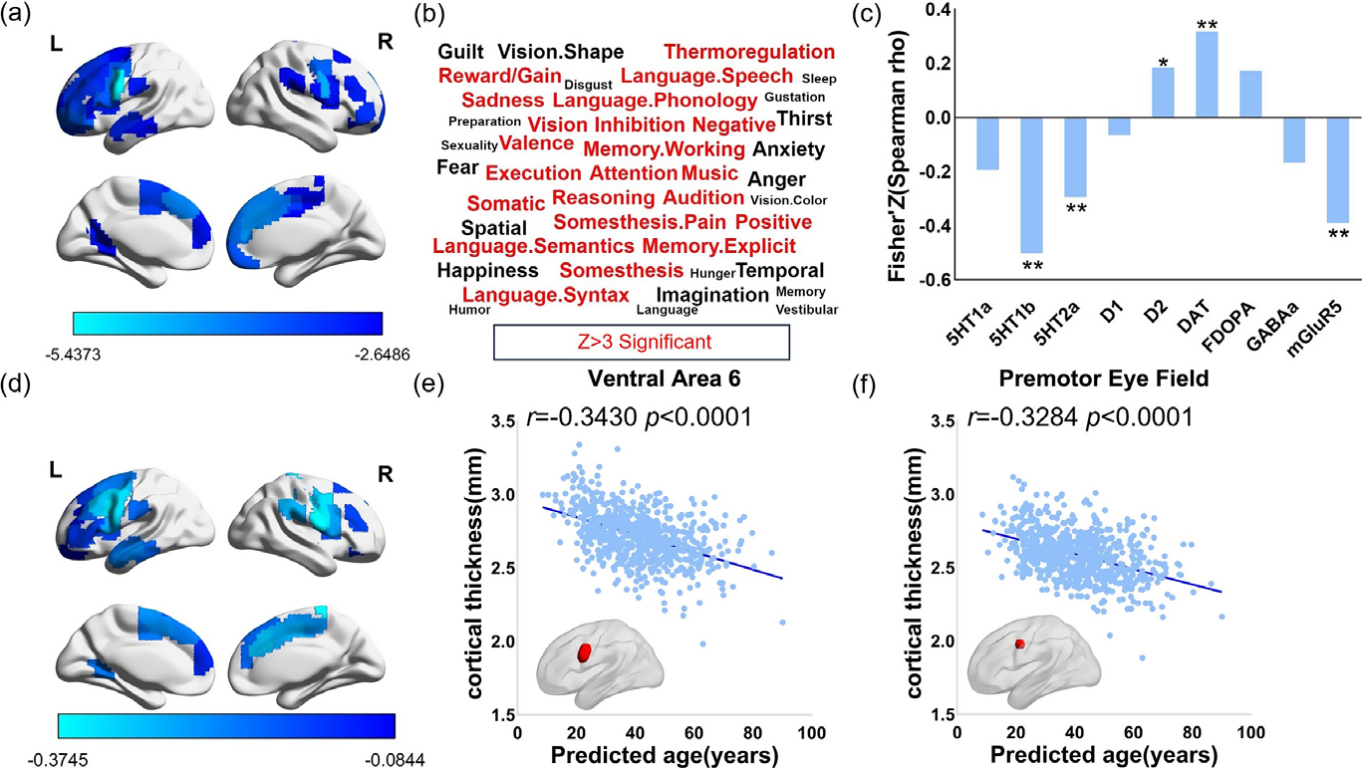
Association between CT alternation in MDD patients and cognitive terms, neurotransmitter receptors/transporters, and CT difference between MDD patients and healthy controls. (a) Brain regions with significant CT reduction (*q* < 0.05, FDR corrected) in the MDD patients. (b) Cognitive words including “Attention”, “Memory working”, “Reasoning”, “Inhibition” were great significant associated with significant *t*-map. (c) Correlation between significant *t*-map of MDD-related CT reduction and neurotransmitter. (d) Brain regions with a significant negative correlation between cortical thickness and predicted age in (a). (e) (f) The partial Pearson correlation coefficients controlling for sex, site, and age between predicted age and cortical thickness were found to be significant in ventral area 6 and PEF. *, *p* < 0.05, **, *p* < 0.01.

After harmonizing individual CT maps from the five sites, we compared ROI-based CT differences (*q* < 0.05, FDR corrected) between MDD patients and all the healthy controls controlling for the effects of age and sex. Compared with healthy controls, MDD patients mainly showed reduced CT in the ventral area and premotor eye field (PFE) in the HCP atlas in the left hemisphere (specific brain regions are provided in Supplementary Table 3, Figure 3a). A significant correlation (*r* = 0.13, *p* = 0.01) was observed between the beta of cortical thickness features in the prediction model and the *t*- map. Additionally, 14 brain regions overlapped between the regions showing significant cortical thinning in MDD patients and the top 60 feature regions in the SVR model (Supplementary Table 8). Furthermore, we employed the permutation-based feature importance method to re-prioritize the significance of features, leading to the identification of overlapping regions that align closely with the results presented earlier (Supplementary Table 10). In the majority of brain regions exhibiting significant differences in cortical thickness between individuals with MDD and healthy controls, the partial Pearson correlation coefficients controlling for sex, site, and age between predicted age and cortical thickness were found to be significant (ventral area 6: *r* = −0.34, *p* < 0.001; PFE: *r* = −0.33, *p* < 0.001. Figure 3d–f, Supplementary Table 3). Notably, the reliability and reproducibility of CT differences between MDD patients and healthy controls are important for the association analysis with gene expression. We used two methods, Combat and regression diagnosis, to obtain overall CT differences between MDD patients and healthy controls (results of regression diagnosis are presented in Supplementary Result 2). To test the consistency of the results obtained from these two methods, between *t*-statistic values of CT differences computed by the two methods, we found a high correlation (*r* = 0.8944, *p* < 0.001, Supplementary Figure 2). In the further analysis we used the *t*-statistic maps calculated by combat harmonization.

### Correlations between cortical thickness alteration in MDD and related cognitive terms

In order to further elucidate the cognitive relevance of cortical thickness changes in these brain regions exhibiting significant differences in cortical thickness between individuals with MDD and healthy controls (Figure 3a), we proceeded to decode these regions. Cognitive terms such as attention, working memory, reasoning, and inhibition were significantly associated with these brain regions (Figure 3b, specific information on cognitive words was included in Supplementary Table 4).

### Relationship between cortical thickness alteration in MDD and neurotransmitter receptors/transporters

Results of correlational analyses between the significant *t*-map (Figure 3a) and density of neurotransmitter receptors/transporters in the human brain showed a significant positive correlation to both the dopaminergic (D2: *r* = 0.18, *p* = 0.0418) and transporter (DAT: *r* = 0.32, *p* = 0.0004), and negative correlation to the serotonergic (5HT1b: *r* = −0.50, *p* = 0.0006; 5HT2a: *r* = −0.30, *p* = 0.0010) systems and glutamatergic (mGluR5: *r* = −0.3903, *p* = 0.0066) receptor (Figure 3c).

### Gene expression profiles related to cortical thickness alteration in MDD

**Figure 4.**
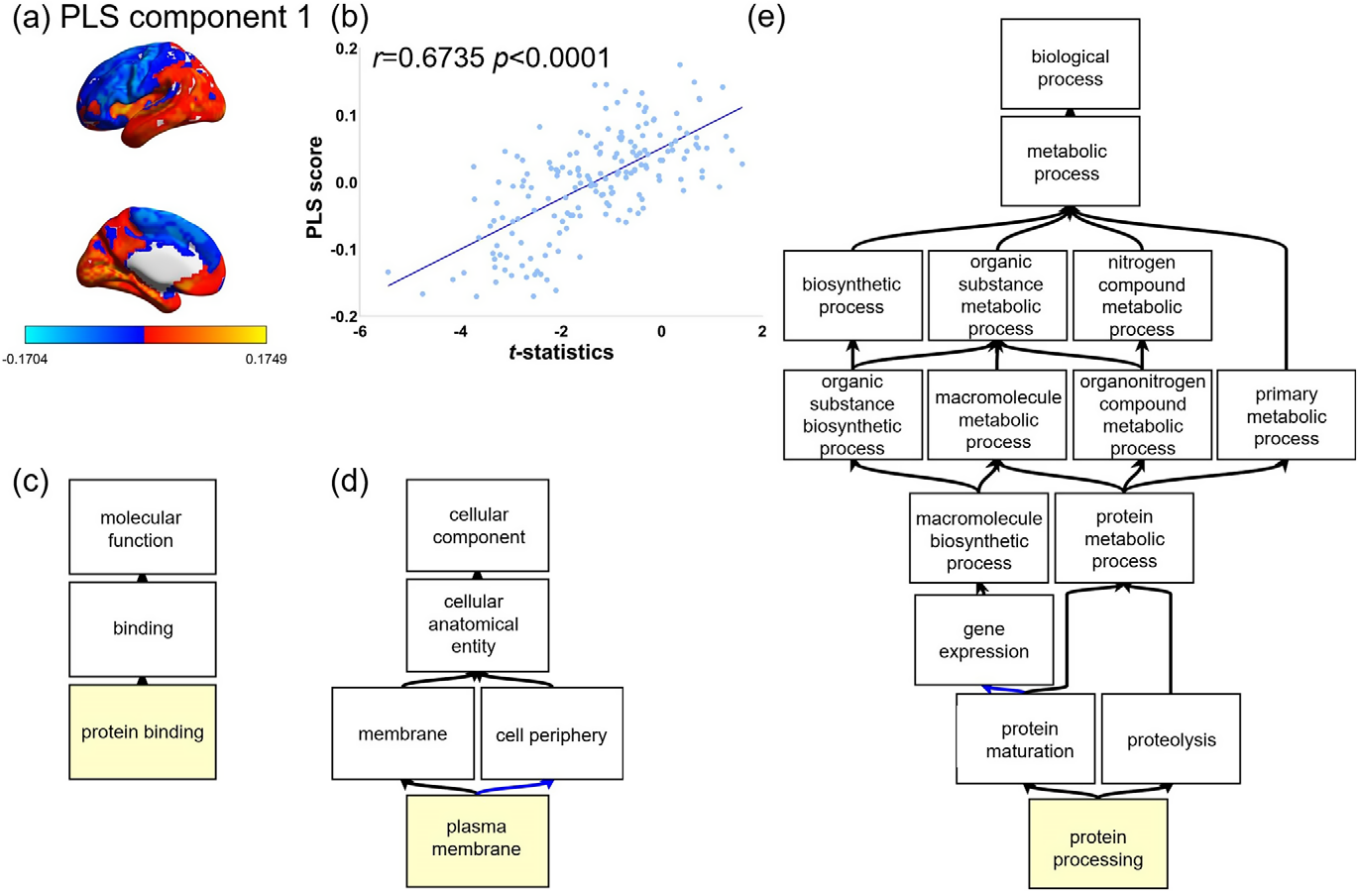
Association between CT alternation in MDD patients and gene expressions. (a) A gene expression profile was identified by the first PLS component. (b) The transcriptional profiles were positively correlated with the between-group *t*-map of the CT differences. (c) MDD-related genes ranked in descending order of the PLS component 1 weight were enriched in the molecular function of protein binding, (d) the cellular component of the plasma membrane, and (e) the biological process of protein processing (FDR *q* < 0.05). The black arrow represents “is a”. The blue arrow represents “part of”.

We obtained normalized expression data of 10027 genes for 176 ROIs of HCP atlas from the AHBA data, these expression data were set as the predictor variables, and 176 ROI’s *t*-statistic of between-group difference of CT was set as the response variable in PLS. The first component of the PLS regression explained 45.22% of the variance in the MDD-related alterations in CT (*p* < 0.05 for component 1, permutation tests with spatial autocorrelation corrected). The first component represented a transcriptional profile with high expression mainly in the parahippocampal area1, piriform cortex, middle temporal area, and presubiculum in the left hemisphere in the HCP atlas (Figure 4a). The regional mapping of these components were positively correlated with the *t*-statistics map of the CT between MDD patients and healthy controls (*r* = 0.6735, *p* < 0.0001, Figure 4b). The Gene Ontology enrichment analysis revealed 60 genes, associated with CT alterations in MDD and ranked in descending order of the first component weight, was enriched in molecular function of protein binding, cellular component of the plasma membrane and biological process of protein processing (*p* < 0.001, FDR-BH corrected *q* < 0.05, Figure 4c–e, other results were posted in the supplementary Table 5).

## Discussion

In this study, we report on the degree of brain aging in patients with MDD relative to HCs, along with alterations in cortical thickness associated with MDD-related brain aging and their association with neurotransmitter expression. Specifically, patients with MDD exhibit higher levels of brain aging than HCs and significant reductions in cortical thickness in brain regions such as the left ventral area and left premotor eye field. Cognitive terms such as attention, working memory, reasoning, and inhibition were significantly associated with the brain regions that exhibited these changes. Further analysis revealed that dopaminergic, transporter, serotonergic systems, and the glutamatergic receptor may be involved in cortical thinning in MDD individuals. Furthermore, gene expression related to protein binding, plasma membrane, and protein processing contribute to MDD-related cortical thinning. These findings enhance our understanding of the neurobiological and genetic mechanisms underlying brain aging in patients with MDD and provide theoretical insights for predicting and preventing further deterioration of MDD.

### Faster brain aging in MDD

Our study found that the mean difference in brain-PAD between MDD patients and healthy controls was +2.6 (Cohen’s d = 0.30, 95% CI: 0.12–0.49), which was smaller than the difference reported in an earlier study (Koutsouleris et al., 2014) on a small sample of MDD patients (mean difference = +4.0, N = 104). However, a recent study by Han et al., which used the ENIGMA database and included a much larger sample of 2675 MDD patients (Han et al., 2021), also found a significant increase in brain aging in MDD patients relative to HCs (mean difference = +1.1, Cohen’s d = 0.14, 95% CI: 0.08–0.20). Our study provides further validation of previous research findings, highlighting that the process of brain aging is notably more accentuated in individuals diagnosed with MDD. Furthermore, our model’s mean absolute error (MAE) in the test sample (6.7 years, age range 18–80 years) was higher than that reported by Han et al. (6.6 years, age range 18–75 years) and Koutsouleris et al. (4.6 years, age range 18–65 years). This difference in MAE may be due to the wider age range of our model, as previous studies have shown that the accuracy of brain age prediction decreases with a wider age range (Han et al., 2021; Koutsouleris et al., 2014). These findings underscore the importance of incorporating ethnic diversity in research on brain aging in individuals with MDD. It is important to acknowledge that regardless of ethnic background, individuals with MDD consistently exhibit more pronounced brain aging, indicating a significant phenomenon that transcends ethnicities. Therefore, brain-PAD can serve as a valuable cross-racial tool for preliminary assessment of brain senescence in patients with MDD and can aid clinicians and researchers in evaluating the severity of brain aging in this group, enabling timely interventions to prevent further deterioration.

### Cortical thinning associated with MDD-related brain aging and its relationship with neurotransmitter expression levels

Our study found that patients with MDD had significantly lower cortical thickness in the left ventral area, and left premotor eye field than HCs. Furthermore, predicted brain age was negatively correlated with cortical thickness in these significant regions, which is consistent with existing literature supporting the importance and sensitivity of cortical thickness to aging (Wang et al., 2014). Spatial correlation analysis revealed significant reductions in the expression of 5HT1b, 5HT2a, and mGluR5 in brain regions showing notable cortical thinning associated with MDD. Additionally, a significant increase in the expression of D2 and DAT was observed. Previous research has implicated a deficiency in monoamine levels, specifically those of serotonin (5-HT), norepinephrine, and dopamine, as one of the biological mechanisms underlying MDD (Fries et al., 2023). Furthermore, a substantial body of evidence supports the association between decreased serotonergic neurotransmission and the occurrence of MDD (Elhwuegi, 2004). While there is limited research specifically addressing the expression levels of mGluR5 in MDD, a study investigating synaptic dysfunction in AD revealed disrupted signaling of mGluR5. It is plausible that the observed decrease in mGluR5 expression in MDD may be associated with the further occurrence of AD, given the potential link between MDD-related brain aging and AD (Abd-Elrahman & Ferguson, 2022). D2 receptors are inhibitory receptors for dopamine neurotransmission. The simultaneous increase in the expression of D2 receptors and DAT in patients with MDD may potentially lead to further suppression of dopamine release. The alterations of D2 receptors and DAT in MDD are closely associated with the onset and progression of the disorder. While the association between D2 receptors, DAT, and depression is well established, further research is needed to explore this relationship in greater depth (Mallet et al., 2019). It is postulated that the expression of these neurotransmitters may potentially contribute to the occurrence of MDD by influencing cortical thickness in the brain.

### Gene ontological basis of cortical thinning associated with MDD-related brain aging

Han et al. reported that age-related structural changes in MDD can be explained by common underlying genetic mechanisms involving brain development and plasticity as well as psychiatric disorders (Han et al., 2021). Exploring the complex relationship between changes in cortical thickness in MDD and gene expression data from the AHBA, we observed that gene expression was the strongest in the parahippocampal area, piriform cortex, middle temporal area, and presubiculum in the HCP atlas, which was highly correlated with decreased cortical thickness in MDD patients. This suggests that the reduced cortical thickness in these regions might be attributable to the higher expression of specific genes. Our connectome-transcriptome association analysis established an association between MDD-related alteration in cortical thickness and MDD-related gene expression enriched in protein binding, plasma membrane, and protein processing. Protein processing involves the synthesis, folding, modification, and degradation of proteins, which are all essential for maintaining cellular homeostasis. Dysregulation in protein processing pathways could result in the accumulation of misfolded or aggregated proteins, leading to cellular stress and dysfunction. These disruptions may contribute to the development of MDD (Soto & Estrada, 2008). In conclusion, our study provides preliminary insights into the possible genetic factors associated with the reduction of cortical thickness in patients with MDD.

### Limitations and future research

Our predictions of brain age in patients with MDD yielded results that are broadly consistent with those of existing studies using other biological markers to predict brain age in this group. However, we must acknowledge several limitations in our study. First, while our study is a cross-sectional study, brain aging is a longitudinal process and the incidence and severity of MDD may change with aging (Berk et al., 2023; Kong, Zhang, Liu, & Pu, 2022; Wu et al., 2019), this necessitates the use of longitudinal data to further explore whether brain-PAD can serve as a marker for early identification of individuals at risk for MDD. Secondly, with regards to the limitations associated with the database utilized in this study: initially, although the inclusion of adolescents and individuals from other East Asian ethnicities in our study is crucial for enhancing the generalizability of our findings, the data utilized in our study originated from an open database from Japan that lacks specific information concerning adolescents and individuals from other East Asian ethnicities. We remain hopeful that future research endeavors will be able to address this gap and contribute to a more comprehensive understanding of the subject matter; furthermore, the limited number of HCs may result in a scenario during SVR training where the number of training samples is less than the number of features, potentially leading to overfitting. While we have included all healthy individuals in the training set to validate the reliability of our findings (Supplementary Results 5), future research will necessitate a larger sample size of individuals from Asian populations. Third, there are potential confounding variables that may impact the accuracy of the results, such as comorbidities associated with MDD, medication use, and demographic factors. However, these variables were not accessible in the public database we utilized. Therefore, in future research, controlling for these factors could be instrumental in validating the reliability of the findings. Fourth, the *t*-map of differential cortical thickness is an indirect indicator reflecting the contribution of each brain region to brain aging. A more intuitive measure of the contribution to brain aging is the beta value in SVR. However, most brain age prediction models are constructed using healthy individuals (Han et al., 2021; Luo et al., 2022), and the beta values in these models represent the normal developmental trajectory. Therefore, future research may need to explore more intuitive indicators that directly reflect the contribution of each brain region to brain aging. Fifth, AD may be one of the consequences of MDD. Therefore, it is worth exploring whether MDD-related brain aging and cortical thinning constitute a risk factor for AD. Additionally, in previous studies, cognitive impairments have been observed in patients with MDD (Bora, Harrison, Yücel, & Pantelis, 2013; Lee, Hermens, Porter, & Hodge, 2012; Nikolin et al., 2021; Rock, Roiser, Riedel, & Blackwell, 2014; Snyder, 2013). However, in the present behavioral correlational analysis, we only explored the brain regions that showed changes in cortical thickness associated with MDD-related brain aging and investigated their relationship with cognitive function. As we used publicly available databases in current research, we lacked access to behavioral measures, which prevented further analysis based on cognitive indicators. Finally, regarding the genetic analysis aspect, the AHBA gene database yielded gene expression data from only six Caucasian participants who did not have MDD; furthermore, for a proportion of them, only left hemisphere data were available. Therefore, larger samples of whole-brain gene expression data from MDD patients of East Asian origin are required to validate the reproducibility of our results.

## Conclusions

Overall, this study demonstrates that brain aging in individuals with MDD is significantly more rapid than in healthy individuals. Furthermore, in the brain regions showing significant differences in cortical thickness between patients with MDD and HCs, brain aging was found to consistently coexist with cortical thinning. These regions are primarily associated with higher-order cognitive terms, including attention, working memory, reasoning, and inhibition. Importantly, they are also significantly correlated with neurotransmitters, such as the dopaminergic receptor, that have close ties to the biological underpinnings of MDD. Connectome-transcriptome association analysis in our study revealed an association between cortical thickness in MDD and protein processing. These findings deepen our understanding of the neurobiological, molecular, and genetic foundations of brain aging in individuals with MDD, providing insights for future research on the underlying mechanisms and strategies for the prevention and management of further deterioration in MDD.

## Supporting information

Dai et al. supplementary material Dai et al. supplementary material

## Supplementary material

The supplementary material for this article can be found at http://doi.org/10.1017/S0033291725000418.
