# Supplementary material for: Accelerated brain aging in patients with major depressive disorder and its neurogenetic basis: evidence from neurotransmitters and gene expression profiles
Source: Psychol Med. 2025 Mar 5;55:e71. doi: 10.1017/S0033291725000418 (PMC12080649; doi:10.1017/S0033291725000418)
Supplement: Dai et al. supplementary material [file S0033291725000418sup001.docx]

*Supplementary*

Accelerated brain aging in patients with major depressive disorder and its neurogenetic basis: evidence from neurotransmitters and gene expression profiles

Dai Haowei^1^, Niu Lijing^1^, Peng Lanxin^1^, Li Qian^1^, Zhang Jiayuan^1^, Chen Keyin^1^, Wang Xingqin^2^, Huang Ruiwang^3^, Lee M.C. Tatia ^4,5,6^, Zhang Ruibin^1,6,7*^

^1^ Laboratory of Cognitive Control and Brain Healthy, School of Public Health, Southern Medical University, Guangzhou, PRC China

^2^ Department of Neurosurgery, Institute of Brain Diseases, Nanfang Hospital of Southern Medical University, Guangzhou, PRC China

^3^School of Psychology, South China Normal University, Guangzhou, China.

^4^State Key Laboratory of Brain and Cognitive Sciences, The University of Hong Kong, Hong Kong, SAR China

^5^Laboratory of Neuropsychology and Human Neuroscience, The University of Hong Kong, Hong Kong, SAR China

^6^Guangdong-Hong Kong-Macao Greater Bay Area Center for Brain Science and Brain-Inspired Intelligence, Guangdong-Hong Kong Joint Laboratory for Psychiatric Disorders, Guangdong Basic Research Center of Excellence for Integrated Traditional and Western Medicine for Qingzhi Diseases

^7^Department of Psychiatry, Zhujiang Hospital, Southern Medical University, Guangzhou, PRC China

*** Corresponding author:**

Ruibin Zhang, Department of Psychology, School of Public Health, Southern Medical University. Email: [ruibinzhang@foxmail.com](mailto:ruibinzhang@foxmail.com)

**Sup Method 1. Spatial correlation between MDD-related alteration in cortical thickness and neurotransmitter receptors/transporters.**

All provided files and the selected PET/SPECT maps are loaded into the atlas space as mean value per file and region. A Spearman’s rank partial correlation analysis is then preformed between the selected receptor/transporter densities maps and CT difference *t*-map between MDD patients and healthy controls depending on the default settings (controlling for partial volume effects and spatial autocorrelation using underlying gray matter probability). For correlation analyses, Fisher's *z*-transformed coefficients are provided as well as the original correlation coefficients. Then one-sample *t*-tests was used to compare the distribution of Fisher's *z* transformed correlation coefficients against null distribution.

**Sup Method 2. Preprocessed gene expression data.**

Gene expression data were downloaded from the Allen Human Brain Atlas (AHBA), which were collected from the brains of six adult donors. Considering that only two donors had tissue samples of the right hemisphere, while six donors had the left hemisphere, we only conducted gene expression analysis within the cortical regions in the left hemisphere. We preprocessed the gene expression microarray data according to a recommended pipeline. The basic workflow involves: (1) confirming and updating probe-to-gene annotations; (2) data filtering: expression values that do not exceed background are removed; (3) probe selection: selection for genes indexed by multiple probes, involves selecting a single representative measure to represent the expression of that gene across all donor brains; (4) sample assignment: tissue samples from the AHBA are mapped to specific brain regions in an imaging dataset; (5) normalization of expression measures to account for inter-individual differences and outlying values; (6) gene-set filtering: remove genes that are inconsistently expressed across six brains and/or select genes in a hypothesis-driven way. The application of these six steps results in a gene expression data matrix that can be used for further analyses (Arnatkevic̆iūtė et al., 2019). Finally, we removed 3 missing ROI lacking gene expression from our analysis including retroinsular cortex, area PGs area V6A and area frontal opercular 5 in HCP atlas.

**Sup Method 3. Mapped MDD risk loci to genes**

We utilized the UCSC Genome Browser with the UCSC hg19/NCBI build 37 position to identify the genes surrounding the MDD-related risk loci. These genes located in the vicinity of the MDD-related risk loci were subsequently considered as MDD-related genes. Boundaries of each gene were expanded by 200 kb (kilobase pair) on each side prior to intersection to capture putative regulatory elements. Where the region does not contain a gene, we listed the nearest gene within 500 kb or 1000 kb.

**Sup Method 4. Gene Ontology (GO) enrichment analysis**

Gene Ontology (GO) enrichment analysis is a statistical method employed within the context of a known set of differentially expressed genes or a specific gene set. It aims to assess whether the distribution of these genes within the GO classification system deviates significantly from the expected pattern. This analysis helps identify which GO terms exhibit a significantly higher frequency than expected by chance within the gene set under investigation. In current study, we seek to explore the regions associated with cortical thickness changes related to brain aging in individuals with MDD and we aim to investigate the GO annotations of the MDD gene set that are associated with these cortical thickness changes. GO classification system comprises three ontologies, namely molecular function, cellular component, and biological process. Each of these ontologies describes different aspects of gene attributes, with the term being the fundamental unit within GO, representing a specific attribute. Finally, Directed Acyclic Graphs, illustrating the hierarchical relationships between GO terms, are presented. These relationships are unidirectional, and there are three types of relationships among GO terms: is a, part of, and regulates. In these figures, GO terms positioned higher in the graph represent more general categories, while those branching downward represent annotations at more specific levels. The color intensity corresponds to the significance of the associated GO term, with darker shades indicating a higher level of significance.

**Sup Table 1. Demographic characteristics of participants included in the sites and datasets.**

| Hub Institute | Site(scanner) | Site Label | HC (No., M:W  Age, y) | MDD (No., M:W  Age, y) |
| --- | --- | --- | --- | --- |
| Hiroshima University | Center of Innovation at Hiroshima univ (VerioDot, Siemens) | COI | 124, 46:78  51.9 (13.4) | 71, 31:40  45.2 (12.5) |
|  | Hiroshima univ hospital (Signa HDxt, GE) | HUH | 67, 29:38  34.7 (13.0) | 57, 32:25  43.3 (12.2) |
|  | Hiroshima Rehabilitation Center (Signa HDxt, GE) | HRC | 49, 13:36  41.7 (11.7) | 16, 6:10  40.5 (11.5) |
|  | Hiroshima Kajikawa Hospital (Spectra, Siemens) | HKH | 29, 12:17  45.4 (9.5) | 33, 20:13  44.8 (11.5) |
| University of Tokyo | Univ of Tokyo (MR750W, GE) | UTO | 170, 78:92  35.6 (17.5) | 62, 36:26  38.7 (11.6) |

**Note:** Data are shown as means (standard deviation). M, men; W, women; HC, healthy controls; MDD, major depressive disorder.

**Sup Table 2. Imaging protocols for structural MRI in the SRPBS Multi-disorder Connectivity and MRI Datasets.**

| Site | COI | HUH | HRC | HKH | UTO |
| --- | --- | --- | --- | --- | --- |
| MRI scanner | SIEMENS | GE | GE | SIEMENS | GE |
|  | Verio.Dot | Sigma HDxt | Sigma HDxt | Spectra | MR750w |
| FoV, mm | 256 | 256 | 256 | 256 | 240 |
| Matrix | 256*256 | 256*256 | 256*256 | 256*256 | 256*256 |
| Voxel size, $\boldsymbol{mm}^{\boldsymbol{3}}$ | 1*1*1 | 1*1*1 | 1*1*1 | 1*1*1 | 1*1*1.2 |
| TR, ms | 2300 | 6812 | 6812 | 1900 | 7.7 |
| TE, ms | 2.98 | 1896 | 1896 | 2.38 | 3.1 |
| TI, ms | 900 | 450 | 450 | 900 | 400 |
| Flip angle, deg | 9 | 20 | 20 | 10 | 11 |

**Note:** FoV: Field of view; TR, repetition time; TE, echo time; TI, inversion time.

**Sup Table 3. Brain regions exhibiting significant differences in cortical thickness between individuals with MDD and healthy controls.**

| Basic Information In HCP-MMP1 Atlas | | | Statistical Information | | |
| --- | --- | --- | --- | --- | --- |
|  |  |  | *t*-statistic  (FDR<0.05, *p*<0.0083) | **CT PA partial corr** | |
| ID | Abbr | Area |  | *r* | *p* (significant<0.0334 FDR<0.05) |
| Left hemisphere | | | | | |
| 10 | FEF | Frontal Eye Fields | -3.29 | -0.30 | 2.78E-15 |
| 11 | PEF | Premotor Eye Field | -4.75 | -0.33 | 0.00E+00 |
| 12 | 55b | Area 55b | -3.68 | -0.34 | 0.00E+00 |
| 26 | SFL | Superior Frontal Language Area | -2.98 | -0.15 | 1.06E-04 |
| 31 | POS1 | Parieto-Occipital Sulcus Area 1 | -2.98 | -0.08 | 2.92E-02 |
| 41 | 24dv | Ventral Area 24d | -3.33 | -0.23 | 2.53E-09 |
| 43 | SCEF | Supplementary and Cingulate Eye Field | -3.31 | -0.25 | 3.52E-11 |
| 56 | 6v | Ventral Area 6 | -5.44 | -0.34 | 0.00E+00 |
| 63 | 8BM | Area 8BM | -4.15 | -0.23 | 1.40E-09 |
| 67 | 8Av | Area 8Av | -2.77 | -0.24 | 2.56E-10 |
| 68 | 8Ad | Area 8Ad | -3.21 | -0.15 | 6.58E-05 |
| 69 | 9m | Area 9 Middle | -2.85 | -0.13 | 6.12E-04 |
| 70 | 8BL | Area 8B Lateral | -3.32 | -0.14 | 3.57E-04 |
| 73 | 8C | Area 8C | -3.08 | -0.32 | 1.11E-16 |
| 74 | 44 | Area 44 | -3.63 | -0.17 | 1.09E-05 |
| 75 | 45 | Area 45 | -2.79 | -0.16 | 4.74E-05 |
| 77 | a47r | Area anterior 47r | -3.72 | -0.07 | 8.99E-02 |
| 78 | 6r | Rostral Area 6 | -3.35 | -0.28 | 2.07E-13 |
| 80 | IFJp | Area IFJp | -3.93 | -0.36 | 0.00E+00 |
| 83 | p9-46v | Area posterior 9-46v | -3.27 | -0.21 | 7.27E-08 |
| 84 | 46 | Area 46 | -2.97 | -0.10 | 1.23E-02 |
| 85 | a9-46v | Area anterior 9-46v | -3.16 | -0.03 | 3.90E-01 |
| 86 | 9-46d | Area 9-46d | -3.28 | -0.04 | 2.97E-01 |
| 87 | 9a | Area anterior 10p | -2.65 | -0.04 | 3.00E-01 |
| 89 | a10p | Area anterior 10p | -3.74 | 0.02 | 5.34E-01 |
| 91 | 11l | Area 11l | -3.24 | -0.10 | 1.10E-02 |
| 96 | 6a | Area 6 anterior | -2.69 | -0.23 | 1.16E-09 |
| 98 | s6-8 | Superior 6-8 Transitional Area | -3.12 | -0.16 | 2.66E-05 |
| 108 | FOP4 | Frontal OPercular Area 4 | -2.96 | -0.17 | 1.21E-05 |
| 121 | ProS | ProStriate Area | -2.67 | -0.23 | 3.69E-09 |
| 128 | STSda | Area STSd anterior | -3.11 | -0.22 | 6.67E-09 |
| 147 | PFop | Area PF opercular | -2.95 | -0.21 | 5.55E-08 |
| 170 | p10p | Area posterior 10p | -2.77 | 0.03 | 3.89E-01 |
| 171 | p47r | Area posterior 47r | -3.41 | -0.15 | 7.04E-05 |
| 176 | STSva | Area STSv c | -2.76 | -0.25 | 1.17E-10 |
| 177 | TE1m | Area TE1 Middle | -2.74 | -0.21 | 7.55E-08 |
| Right hemisphere | | | | | |
| 10 | FEF | Frontal Eye Fields | -3.72 | -0.31 | 2.22E-16 |
| 12 | 55b | Area 55b | -3.70 | -0.34 | 0.00E+00 |
| 40 | 24dd | Dorsal Area 24d | -2.71 | -0.26 | 9.36E-12 |
| 41 | 24dv | Ventral Area 24d | -3.31 | -0.29 | 2.75E-14 |
| 53 | 3a | Area 3a | -2.78 | -0.37 | 0.00E+00 |
| 56 | 6v | Ventral Area 6 | -4.52 | -0.35 | 0.00E+00 |
| 57 | p24pr | Area Posterior 24 prime | -2.74 | -0.05 | 1.75E-01 |
| 60 | p32pr | Area p32 prime | -4.03 | -0.29 | 3.99E-14 |
| 62 | d32 | Area dorsal 32 | -4.29 | -0.21 | 6.53E-08 |
| 63 | 8BM | Area 8BM | -4.11 | -0.20 | 1.15E-07 |
| 66 | 47m | Area 47m | -2.69 | -0.13 | 7.68E-04 |
| 69 | 9m | Area 9 Middle | -3.92 | -0.08 | 3.72E-02 |
| 72 | 10d | Area 10d | -3.17 | 0.00 | 9.04E-01 |
| 78 | 6r | Rostral Area 6 | -2.79 | -0.27 | 1.16E-12 |
| 80 | IFJp | Area IFJp | -2.88 | -0.28 | 3.54E-13 |
| 83 | p9-46v | Area posterior 9-46v | -2.96 | -0.16 | 2.02E-05 |
| 88 | 10v | Area 10v | -3.59 | -0.03 | 5.05E-01 |
| 90 | 10pp | Polar 10p | -3.29 | -0.07 | 9.16E-02 |
| 98 | s6-8 | Superior 6-8 Transitional Area | -2.87 | -0.16 | 3.23E-05 |
| 99 | 43 | Area 43 | -2.87 | -0.21 | 8.38E-08 |
| 114 | FOP3 | Frontal OPercular Area 3 | -2.78 | -0.18 | 5.01E-06 |
| 148 | PF | Area PF Complex | -2.76 | -0.29 | 9.44E-15 |
| 171 | p47r | Area posterior 47r | -3.21 | -0.06 | 1.30E-01 |
| 179 | a32pr | Area anterior 32 prime | -4.25 | -0.31 | 1.11E-16 |

**Note:** Abbr: Abbreviation. CT, cortical thickness. PA, predicted age.

**Sup Table 4. Cognitive terms of brain regions exhibiting significant differences in cortical thickness between individuals with MDD and healthy controls with combat method**

| Category | Domain | *Z*-score |
| --- | --- | --- |
| Execution | Action | 5.315621 |
| Execution.Speech | Action | 1.36007 |
| Imagination | Action | 2.852024 |
| Inhibition | Action | 7.903724 |
| Motor Learning | Action | 0.711485 |
| Observation | Action | 1.681972 |
| Preparation | Action | 2.506103 |
| Attention | Cognition | 9.732361 |
| Language | Cognition | 0.389405 |
| Language.Orthography | Cognition | 2.519995 |
| Language.Phonology | Cognition | 5.977259 |
| Language.Semantics | Cognition | 5.938369 |
| Language.Speech | Cognition | 4.955456 |
| Language.Syntax | Cognition | 3.103498 |
| Memory | Cognition | 1.424871 |
| Memory.Explicit | Cognition | 5.097337 |
| Memory.Implicit | Cognition | 0.575932 |
| Memory.Working | Cognition | 9.624829 |
| Music | Cognition | 6.299099 |
| Reasoning | Cognition | 8.760628 |
| Social Cognition | Cognition | 6.286857 |
| Somatic | Cognition | 3.345105 |
| Spatial | Cognition | 2.82411 |
| Temporal | Cognition | 2.931694 |
| Intensity | Emotion | 0.277358 |
| Negative | Emotion | 5.203478 |
| Anger | Emotion | 2.814719 |
| Anxiety | Emotion | 2.635522 |
| Disgust | Emotion | 1.733655 |
| Embarrassment | Emotion | 0.965957 |
| Fear | Emotion | 1.470352 |
| Guilt | Emotion | 0.781242 |
| Punishment/Loss | Emotion | 2.251061 |
| Sadness | Emotion | 3.298316 |
| Positive | Emotion | 3.217708 |
| Happiness | Emotion | 2.924648 |
| Humor | Emotion | -0.56019 |
| Reward/Gain | Emotion | 3.740461 |
| Valence | Emotion | 4.477303 |
| Baroregulation | Interoception | 0.744065 |
| Gastrointestinal/Genitourinary | Interoception | 1.659084 |
| Heartbeat Detection | Interoception | 0.958457 |
| Hunger | Interoception | 0.166347 |
| Osmoregulation | Interoception | 0.986865 |
| Respiration Regulation | Interoception | 1.061115 |
| Sexuality | Interoception | 1.884503 |
| Sleep | Interoception | -0.06409 |
| Thermoregulation | Interoception | 3.426511 |
| Thirst | Interoception | 1.571892 |
| Vestibular | Interoception | 0.289662 |
| Audition | Perception | 7.266374 |
| Gustation | Perception | 1.964663 |
| Olfaction | Perception | -0.22042 |
| Somesthesis | Perception | 3.962792 |
| Somesthesis.Pain | Perception | 7.69137 |
| Vision | Perception | 5.879891 |
| Vision.Color | Perception | 2.178209 |
| Vision.Motion | Perception | 2.118424 |
| Vision.Shape | Perception | 2.697736 |

**Sup Table 5. Enrichment of MDD-related genes associated with MDD-related alterations in CT and gene expression data in the left hemisphere.**

| Term | function category | ID | Input gene number | Background number | *p* | Corrected *p* | Input Genes |
| --- | --- | --- | --- | --- | --- | --- | --- |
| protein binding | molecular  function | GO:0005515 | 38 | 11779 | 1.02E-07 | 6.38E-05 | HS3ST5\|ACADVL\|SLC7A8\|WASF1\|RCAN3\|CNTN1\|ECE1\|LAMB1\|OXER1\|HSD17B11\|DLG4\|ALCAM\|ASPH\|GABARAP\|DVL2\|NEURL4\|MTA3\|ARHGEF12\|PLCB4\|ZBTB5\|CREB5\|PTGIS\|NEGR1\|TNC\|KCNB1\|PIK3C3\|PSMB5\|PHF23\|GPHN\|TJP2\|IRF2\|NIPAL3\|ETV5\|PIP5K1B\|MCC\|PPIE\|C14orf119\|CDC40 |
| plasma membrane | cellular  component | GO:0005886 | 19 | 4619 | 3.71E-05 | 1.16E-02 | TJP2\|OXER1\|ETV5\|DLG4\|SLC7A8\|MCC\|GRIK4\|GABARAP\|NEGR1\|LAMP5\|CNTN1\|ICAM2\|ECE1\|ASPH\|STEAP1\|KCNB1\|STEAP2\|DGKG\|GPHN |
| protein processing | biological process | GO:0016485 | 3 | 56 | 1.03E-04 | 1.43E-02 | PIK3C3\|CNTN1\|ECE1 |
| cytoplasm | cellular component | GO:0005737 | 18 | 4624 | 1.31E-04 | 1.43E-02 | MTA3\|ARHGEF12\|HSD17B11\|DLG4\|SLC7A8\|CFAP69\|RCAN3\|YBX2\|MCC\|GRHPR\|DVL2\|MTCL1\|NEURL4\|PSMB5\|PIK3C3\|PPIE\|PHF23\|GPHN |
| cytosol | cellular component | GO:0005829 | 19 | 5095 | 1.41E-04 | 1.43E-02 | TJP2\|IRF2\|ARHGEF12\|STEAP2\|HSD17B11\|GABARAP\|EIF4G3\|PIP5K1B\|MCC\|GRHPR\|DGKG\|DVL2\|C14orf119\|ACADVL\|PLCB4\|PSMB5\|PIK3C3\|PPIE\|GPHN |
| lateral plasma membrane | cellular component | GO:0016328 | 3 | 65 | 1.58E-04 | 1.43E-02 | DVL2\|KCNB1\|MTCL1 |
| oxidation-reduction process | biological process | GO:0055114 | 6 | 525 | 1.60E-04 | 1.43E-02 | GRHPR\|HSD17B11\|PTGIS\|STEAP1\|STEAP2\|GPHN |
| uropod | cellular component | GO:0001931 | 2 | 12 | 2.06E-04 | 1.61E-02 | PIP5K1B\|ICAM2 |
| glutamate receptor signaling pathway | biological process | GO:0007215 | 2 | 16 | 3.45E-04 | 2.16E-02 | KCNB1\|GRIK4 |
| regulation of protein secretion | biological process | GO:0050708 | 2 | 16 | 3.45E-04 | 2.16E-02 | PIK3C3\|DLG4 |
| Golgi to plasma membrane transport | biological process | GO:0006893 | 2 | 23 | 6.72E-04 | 3.34E-02 | STEAP2\|DLG4 |
| oxidoreductase activity, acting on the CH-OH group of donors, NAD or NADP as acceptor | molecular function | GO:0016616 | 2 | 24 | 7.27E-04 | 3.34E-02 | GRHPR\|HSD17B11 |
| cell adhesion | biological process | GO:0007155 | 5 | 478 | 8.60E-04 | 3.34E-02 | LAMB1\|ALCAM\|CNTN1\|ICAM2\|TNC |
| smooth endoplasmic reticulum | cellular component | GO:0005790 | 2 | 27 | 9.06E-04 | 3.34E-02 | PLCB4\|GABARAP |
| hippo signaling | biological process | GO:0035329 | 2 | 27 | 9.06E-04 | 3.34E-02 | TJP2\|DVL2 |

**Sup Table 6. Relationship between *t*-map constructed by regression diagnose and neurotransmitter receptors/transporters**

| PETMAP | Fisher‘*Z*(Spearman rho) | *p*_exact(spatial permutations) |
| --- | --- | --- |
| 5HT1a | -0.053 | 0.67846 |
| 5HT1b | -0.3061 | 0.024595 |
| 5HT2a | -0.054 | 0.56749 |
| D1 | 0.0225 | 0.80784 |
| D2 | -0.0643 | 0.4921 |
| DAT | 0.1513 | 0.10438 |
| FDOPA | -0.1362 | 0.13517 |
| GABAa | -1.04E-04 | 0.9992 |
| mGluR5 | -0.0354 | 0.79944 |

**Sup table 7. Cognitive terms of brain regions exhibiting significant differences in cortical thickness between individuals with MDD and healthy controls with regression diagnose method**

| Category | Domain | *Z*-score |
| --- | --- | --- |
| Execution | Action | 7.521223 |
| Execution.Speech | Action | 2.198396 |
| Imagination | Action | 3.34468 |
| Inhibition | Action | 2.917369 |
| Motor Learning | Action | 0.633341 |
| Observation | Action | 1.315688 |
| Preparation | Action | 1.270317 |
| Attention | Cognition | 7.289209 |
| Language | Cognition | 3.063137 |
| Language.Orthography | Cognition | 3.533965 |
| Language.Phonology | Cognition | 5.081442 |
| Language.Semantics | Cognition | 7.448555 |
| Language.Speech | Cognition | 4.041376 |
| Language.Syntax | Cognition | 2.717473 |
| Memory | Cognition | 0.760376 |
| Memory.Explicit | Cognition | 6.201941 |
| Memory.Implicit | Cognition | 1.547812 |
| Memory.Working | Cognition | 5.745253 |
| Music | Cognition | 4.008462 |
| Reasoning | Cognition | 8.348105 |
| Social Cognition | Cognition | 6.042756 |
| Somatic | Cognition | 0.249182 |
| Spatial | Cognition | 2.378884 |
| Temporal | Cognition | 1.456746 |
| Intensity | Emotion | 1.616946 |
| Negative | Emotion | 6.235423 |
| Anger | Emotion | 1.965177 |
| Anxiety | Emotion | 2.149445 |
| Disgust | Emotion | 2.551553 |
| Embarrassment | Emotion | 2.342867 |
| Fear | Emotion | 1.544505 |
| Guilt | Emotion | 1.186173 |
| Punishment/Loss | Emotion | 1.270569 |
| Sadness | Emotion | 2.850024 |
| Positive | Emotion | 3.950027 |
| Happiness | Emotion | 1.499157 |
| Humor | Emotion | -1.231 |
| Reward/Gain | Emotion | 4.508857 |
| Valence | Emotion | 3.735026 |
| Baroregulation | Interoception | 0.478551 |
| Gastrointestinal/Genitourinary | Interoception | 0.703591 |
| Heartbeat Detection | Interoception | 0.199328 |
| Hunger | Interoception | 0.61396 |
| Osmoregulation | Interoception | 1.191296 |
| Respiration Regulation | Interoception | 0.374365 |
| Sexuality | Interoception | 2.120835 |
| Sleep | Interoception | 0.058556 |
| Thermoregulation | Interoception | 2.058811 |
| Thirst | Interoception | 1.075949 |
| Vestibular | Interoception | 1.621606 |
| Audition | Perception | 3.57718 |
| Gustation | Perception | 1.417862 |
| Olfaction | Perception | 1.112475 |
| Somesthesis | Perception | 1.276321 |
| Somesthesis.Pain | Perception | 6.774923 |
| Vision | Perception | 4.97022 |
| Vision.Color | Perception | 2.26921 |
| Vision.Motion | Perception | 1.465523 |
| Vision.Shape | Perception | 2.13255 |

**Sup table 8.** **Overlap of brain regions exhibiting significant differences in cortical thickness and high weight in prediction.**

| Basic Information In HCP-MMP1 Atlas | | | Statistical Information | | |
| --- | --- | --- | --- | --- | --- |
|  |  |  | Beta in trained model |  | |
| ID | Abbr | Area |  | *t* | *p* (significant<0.0334 FDR<0.05) |
| Left hemisphere | | | | | |
| 12 | 55b | Area 55b | -3.68 | -3.68 | 2.50E-04 |
| 43 | SCEF | Supplementary and Cingulate Eye Field | -3.31 | -3.32 | 9.62E-04 |
| 63 | 8BM | Area 8BM | -4.15 | -4.15 | 3.76E-05 |
| 86 | 9-46d | Area 9-46d | -3.28 | -3.28 | 1.11E-03 |
| 91 | 11l | Area 11l | -3.24 | -3.24 | 1.24E-03 |
| 96 | 6a | Area 6 anterior | -2.69 | -2.69 | 7.38E-03 |
| 121 | ProS | ProStriate Area | -2.67 | -2.67 | 7.69E-03 |
| 176 | STSva | Area STSv c | -2.76 | -2.76 | 5.86E-03 |
| Right hemisphere | | | | | |
| 53 | 3a | Area 3a | -2.78 | -2.78 | 5.55E-03 |
| 56 | 6v | Ventral Area 6 | -4.52 | -4.52 | 7.15E-06 |
| 62 | d32 | Area dorsal 32 | -4.29 | -4.29 | 2.05E-05 |
| 63 | 8BM | Area 8BM | -4.11 | -4.11 | 4.43E-05 |
| 72 | 10d | Area 10d | -3.17 | -3.17 | 1.58E-03 |
| 179 | a32pr | Area anterior 32 prime | -4.25 | -4.25 | 2.48E-05 |

**Note:** Abbr: Abbreviation.

**Sup Table 9. Overlap of brain regions exhibiting high weight (regions with** **absolute beta values greater than 6) in prediction in two prediction model (feature matrix with combatHarmonization and feature matrix without combatHarmonization).**

| Basic Information In HCP-MMP1 Atlas | | | \| beta value of two prediction model \| \| --- \| \|  \| | | |
| --- | --- | --- | --- | --- | --- | --- | --- |
| ID | Abbr | Area | | With combat | Without Combat |
| Left hemisphere | | | | | |
| 1 | V1 | Primary Visual Cortex | | -8.46 | -11.33 |
| 5 | V3 | Third Visual Area | | -7.00 | -6.07 |
| 8 | 4 | PriMary Motor Cortex | | -13.72 | -7.57 |
| 13 | V3A | Area V3A | | 8.61 | 6.30 |
| 15 | POS2 | Parieto-Occipital Sulcus Area 2 | | 6.73 | 13.93 |
| 18 | FFC | Fusiform Face Complex | | 6.80 | 7.07 |
| 22 | PIT | Posterior InferoTemporal | | 11.37 | 9.98 |
| 44 | 6ma | Area 6m anterior | | -6.23 | -9.90 |
| 48 | LIPv | Area Lateral IntraParietal ventral | | 8.02 | 6.14 |
| 53 | 3a | Area 3a | | -7.68 | -9.70 |
| 91 | 11l | Area 11l | | 9.70 | 8.88 |
| 97 | i6-8 | Inferior 6-8 Transitional Area | | -8.21 | -6.03 |
| 98 | s6-8 | Superior 6-8 Transitional Area | | -11.98 | -6.29 |
| 107 | TA2 | Area TA2 | | -7.01 | -6.42 |
| 110 | Pir | Pirform Cortex | | 8.41 | 10.57 |
| 116 | PFt | Area PFt | | -9.09 | -10.89 |
| 119 | PreS | PreSubiculum | | -14.15 | -12.32 |
| 121 | ProS | ProStriate Area | | -17.02 | -15.30 |
| 131 | TGd | Area TG dorsal | | 12.97 | 12.54 |
| 132 | TE1a | Area TE1 anterior | | -7.51 | -7.29 |
| 141 | TPOJ3 | Area TemporoParietoOccipital Junction 3 | | 6.01 | 6.32 |
| 154 | VMV3 | VentroMedial Visual Area 3 | | -10.94 | -11.04 |
| 160 | VMV2 | Area 2 | | -10.65 | -6.32 |
| 168 | Ig | Insular Granular Complex | | 9.08 | 6.86 |
| 171 | p47r | Area posterior 47r | | -11.28 | -7.17 |
| 174 | LBelt | Lateral Belt Complex | | -8.19 | -10.43 |
| 178 | PI | Para-Insular Area | | -8.01 | -8.38 |
| Right hemisphere | | | | | |
| 8 | 4 | PriMary Motor Cortex | | -10.16 | -9.08 |
| 11 | PEF | Premotor Eye Field | | -12.34 | -9.35 |
| 12 | 55b | Area 55b | | -8.35 | -7.24 |
| 16 | V7 | Seventh Visual Area | | -7.51 | -8.11 |
| 18 | FFC | Fusiform Face Complex | | 12.48 | 17.53 |
| 21 | LO2 | Area Lateral Occipital 2 | | 10.17 | 7.43 |
| 36 | 5m | Area 5m | | 8.34 | 6.63 |
| 48 | LIPv | Area Lateral IntraParietal ventral | | 12.61 | 9.41 |
| 53 | 3a | Area 3a | | -10.65 | -12.16 |
| 62 | d32 | Area dorsal 32 | | -11.88 | -14.53 |
| 73 | 8C | Area 8C | | -9.30 | -10.18 |
| 84 | 46 | Area 46 | | 7.23 | 7.42 |
| 102 | OP2-3 | Area OP2-3/VS | | 7.63 | 7.88 |
| 125 | A5 | Auditory 5 Complex | | -6.22 | -9.81 |
| 131 | TGd | Area TG dorsal | | 7.01 | 8.77 |
| 139 | TPOJ1 | Area TemporoParietoOccipital Junction 1 | | -6.41 | -6.20 |
| 160 | VMV2 | Area 2 | | -9.72 | -11.25 |
| 163 | VVC | Ventral Visual Complex | | -7.12 | -9.33 |
| 166 | pOFC | posterior OFC Complex | | 8.96 | 6.63 |
| 168 | Ig | Insular Granular Complex | | 7.32 | 6.19 |
| 173 | MBelt | Medial Belt Complex | | -8.67 | -6.94 |
| 179 | a32pr | Area anterior 32 prime | | -7.27 | -8.58 |

**Note:** Abbr: Abbreviation. Combat: Combat Harmonization.

**Sup table 10.** **Overlap of brain regions exhibiting significant differences in cortical thickness and high importance in prediction (produced by permutation-based feature importance method).**

| Basic Information In HCP-MMP1 Atlas | | | Statistical Information | | |
| --- | --- | --- | --- | --- | --- |
|  |  |  | Importance |  | |
| ID | Abbr | Area |  | *t* | *p* (significant<0.0334 FDR<0.05) |
| Left hemisphere | | | | | |
| 12 | 55b | Area 55b | 0.6962 | -3.68 | 2.50E-04 |
| 63 | 8BM | Area 8BM | 0.7009 | -4.15 | 3.76E-05 |
| 86 | 9-46d | Area 9-46d | 0.6992 | -3.28 | 1.11E-03 |
| 89 | a10p | Area anterior 10p | 0.5735 | -3.74 | 2.02E-04 |
| 91 | 11l | Area 11l | 0.6123 | -3.24 | 1.24E-03 |
| 96 | 6a | Area 6 anterior | 0.8116 | -2.69 | 7.38E-03 |
| 121 | ProS | ProStriate Area | 1.0829 | -2.67 | 7.69E-03 |
| 147 | PFop | Area PF opercular | 0.7152 | -2.95 | 3.24E-03 |
| Right hemisphere | | | | | |
| 53 | 3a | Area 3a | 0.7813 | -2.78 | 5.55E-03 |
| 62 | d32 | Area dorsal 32 | 0.14451 | -4.29 | 2.05E-05 |
| 63 | 8BM | Area 8BM | 0.7566 | -4.11 | 4.43E-05 |
| 72 | 10d | Area 10d | 0.6771 | -3.17 | 1.58E-03 |
| 179 | a32pr | Area anterior 32 prime | 0.6032 | -4.25 | 2.48E-05 |

**Note:** Abbr: Abbreviation.

**Sup results 1 Brain age prediction model performance with feature matrix of a2009s cortical thickness atlas**

The correlation between chronological age and predicted age on the test are presented in Sup Fig. 1a and Sup Fig. 1b. The difference of brain-PAD between healthy controls and MDD patients in test sample is significant (*t*=2.26, *p*=0.0245, Sup Fig. 1d). And permutation test for difference of brain-PAD is significant (*p*=0.0050). Compare with healthy controls in test sample, MDD patients shows significant higher predicted age (*p*=0.0197), while there is no difference in chronological age (*p*=0.3955, Sup Fig. 1c).


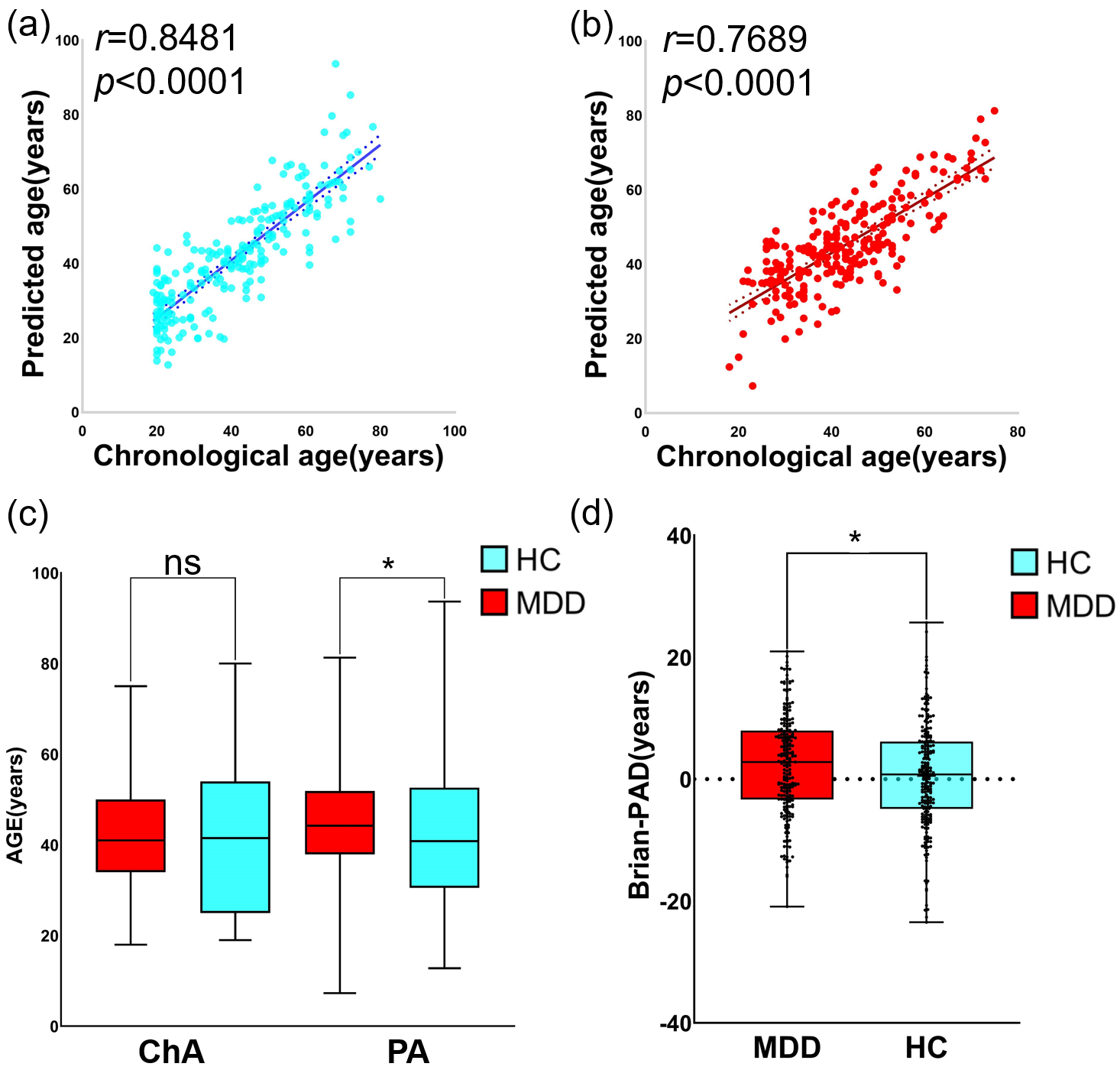


Sup Fig. 1. Performance of brain age prediction with a2009s atlas. (a) Positive correlation between the chronological age and predicted age in the test healthy controls. (b) Positive correlation between the chronological age and predicted age in the test MDD patients. (c) Group comparison between MDD patients and healthy controls in the test dataset and there is no significant difference in chronological age between two groups, while the predicted age in MDD patients is significantly higher than that in healthy controls. (d) Group comparison of the brain-PAD between MDD patients and healthy controls in test dataset and brain-PAD in MDD patients is significantly higher than that in healthy controls. Ns means that the difference between MDD and HC is not significant. *, *p*<0.05.

**Sup result 2 Correlations between *t*-map constructed by regression diagnose and related cognitive terms**

We proceeded to decode significant *t*-map constructed by regression diagnose (Sup Fig. 2a). Cognitive words including reasoning, execution, attention and memory working were significant associated with these brain regions (Sup Fig. 2b, specific information of cognitive word was included in Supplementary table 7).


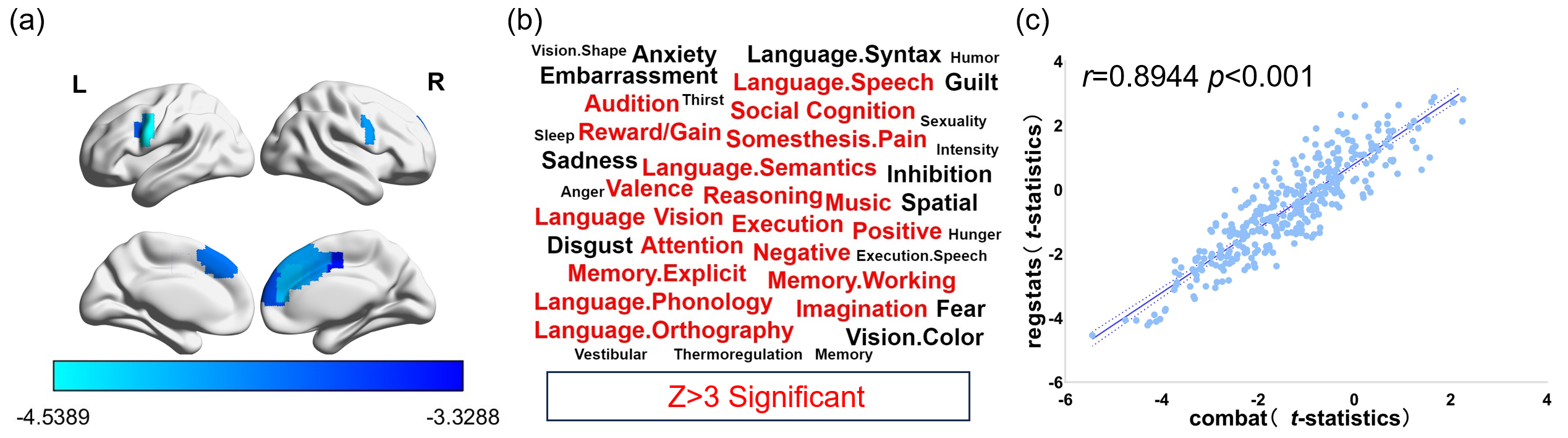


Sup Fig. 2. Association between CT alternation in MDD patients and cognitive terms. (a) Brain regions with significant CT reduction with regression diagnose method (*p*<0.05, FDR corrected) in the MDD patients. (b) Cognitive words including “Reasoning”, “Attention”, “Memory working”, “execution” were great significant associated with the ROI exhibiting significant differences in cortical thickness between individuals with MDD and healthy controls. (c) Scatterplot shows correlation between CT differences derived from harmonization (x-axis) and regression diagnose (y-axis) methods.

**Sup result 3 Gene expression profiles related to *t*-map constructed by regression diagnose**

Gene expression data were set as the predictor variables and 176 ROI’s *t*-map constructed by regression diagnose was set as the response variable in PLS. The first component of the PLS regression explained 40.17% of the variance in the MDD-related alterations in CT (*p*<0.05 for component 1, permutation tests with spatial autocorrelation corrected). The regional mapping of this components was positively correlated with the t-statistics map of the CT between MDD patients and healthy controls (Sup Fig. 3a, Sup Fig. 3b, *r*= 0.6338, *p*<0.0001). The PLS score obtained from two methods showed a high correlation (*r*=0.7113, *p*<0.0001, Sup Fig. 3c).


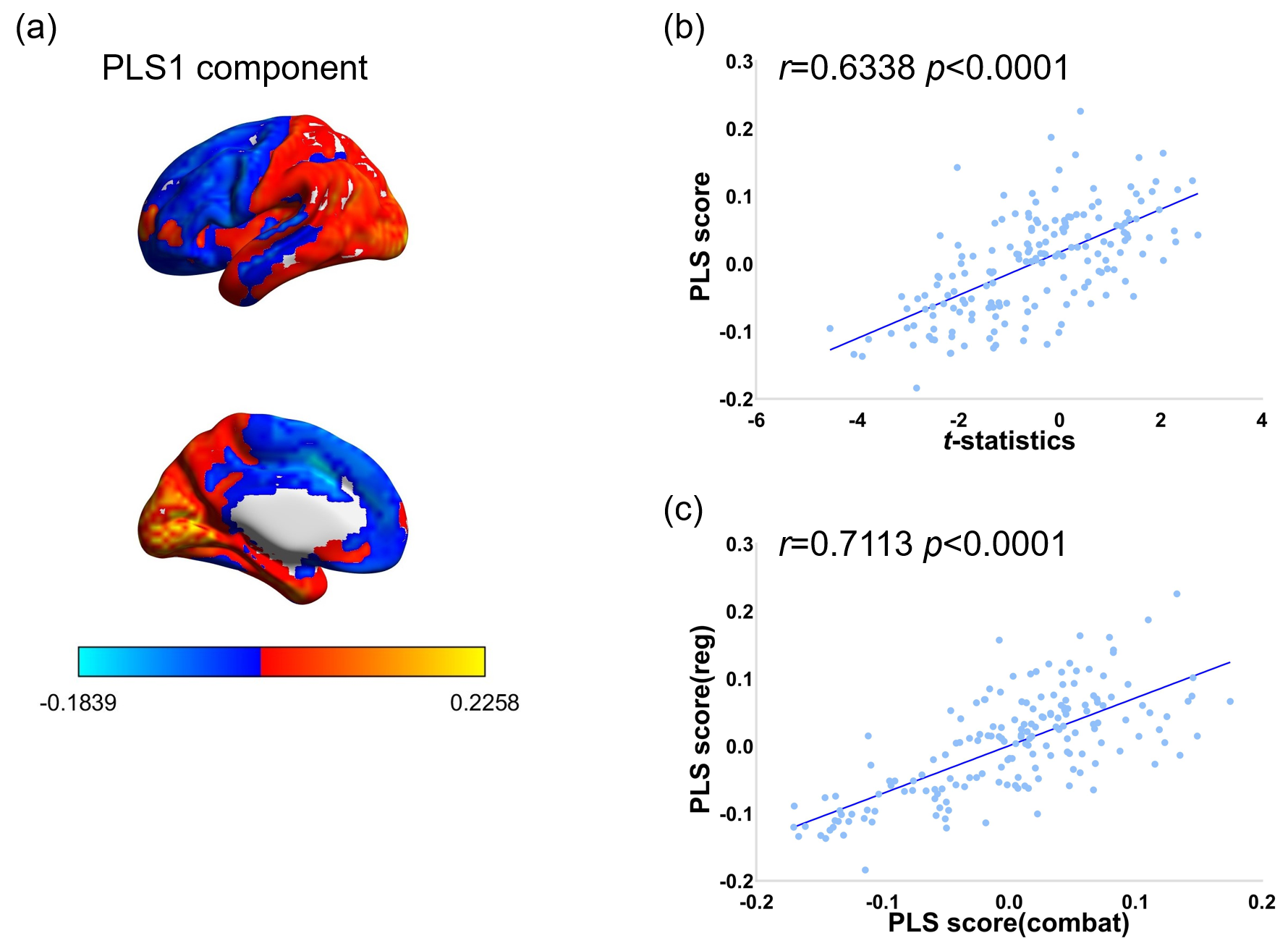


Sup Fig. 3. Association between CT alternation in MDD patients with regression diagnose method and gene expressions. (a) A gene expression profile identified by the first PLS component. (b) The transcriptional profiles were positively correlated with the between-group T-map of the CT differences. (c) PLS score association with *t*-map constructed by combat method and regression diagnose method were significantly positive correlation.

**Sup results 4 Brain age prediction model performance with** **harmonized feature matrix of HCP cortical thickness atlas**

The correlation between chronological age and predicted age on the test are presented in Sup Fig. 4a and Sup Fig. 4b. The difference of brain-PAD between healthy controls and MDD patients in test sample is significant (*t*=2.36, *p*=0.0188, Sup Fig. 4d). And permutation test for difference of brain-PAD is significant (*p*=0.0029). Compare with healthy controls in test sample, MDD patients shows significant higher predicted age (*p*=0.0300), while there is no difference in chronological age (*p*=0.6460, Sup Fig. 4c).


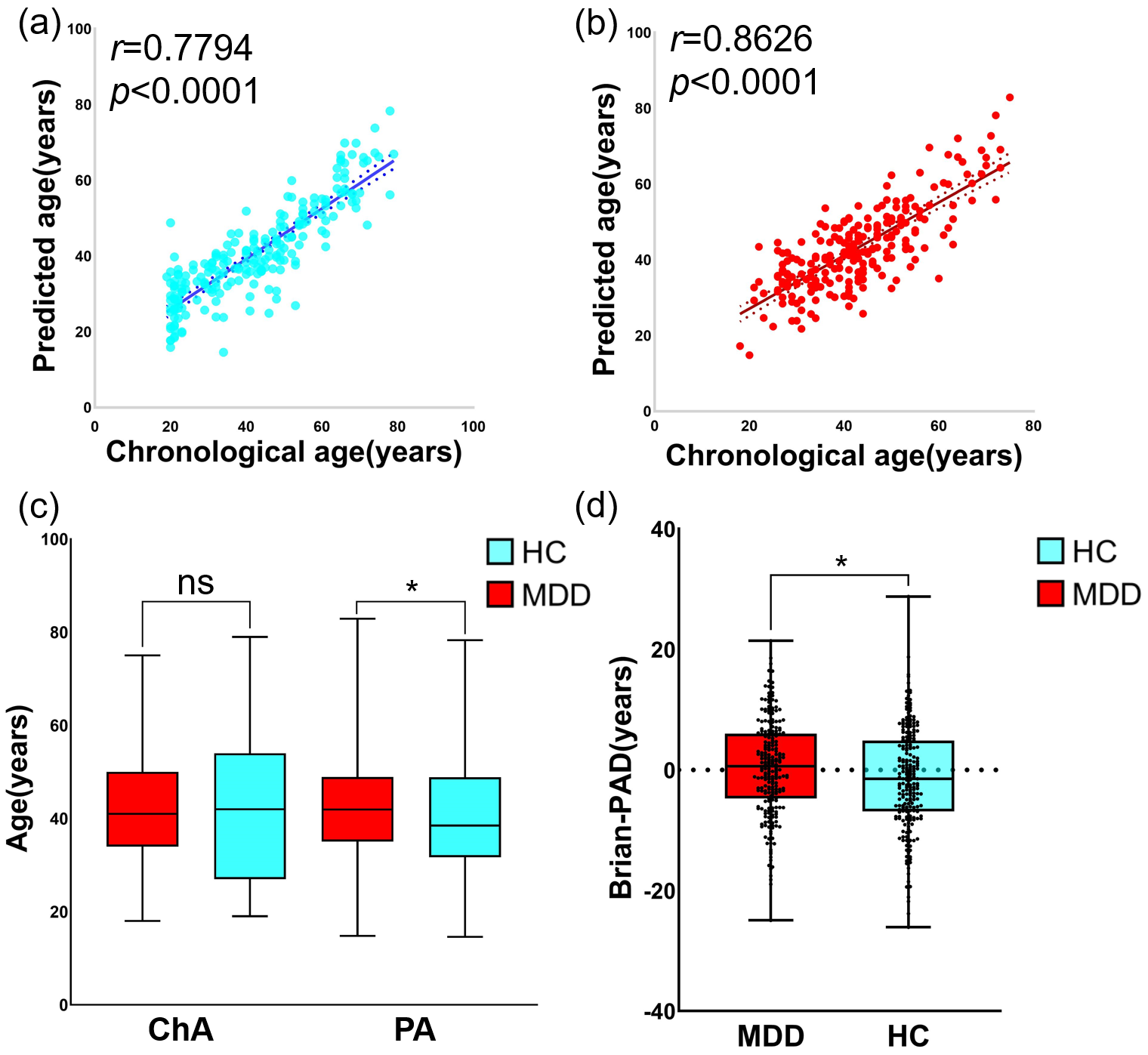


Sup Fig. 4. Performance of brain age prediction with harmonized feature matrix of HCP atlas. (a) Positive correlation between the chronological age and predicted age in the test healthy controls. (b) Positive correlation between the chronological age and predicted age in the test MDD patients. (c) Group comparison between MDD patients and healthy controls in the test dataset and there is no significant difference in chronological age between two groups, while the predicted age in MDD patients is significantly higher than that in healthy controls. (d) Group comparison of the brain-PAD between MDD patients and healthy controls in test dataset and brain-PAD in MDD patients is significantly higher than that in healthy controls. Ns means that the difference between MDD and HC is not significant. *, *p*<0.05.


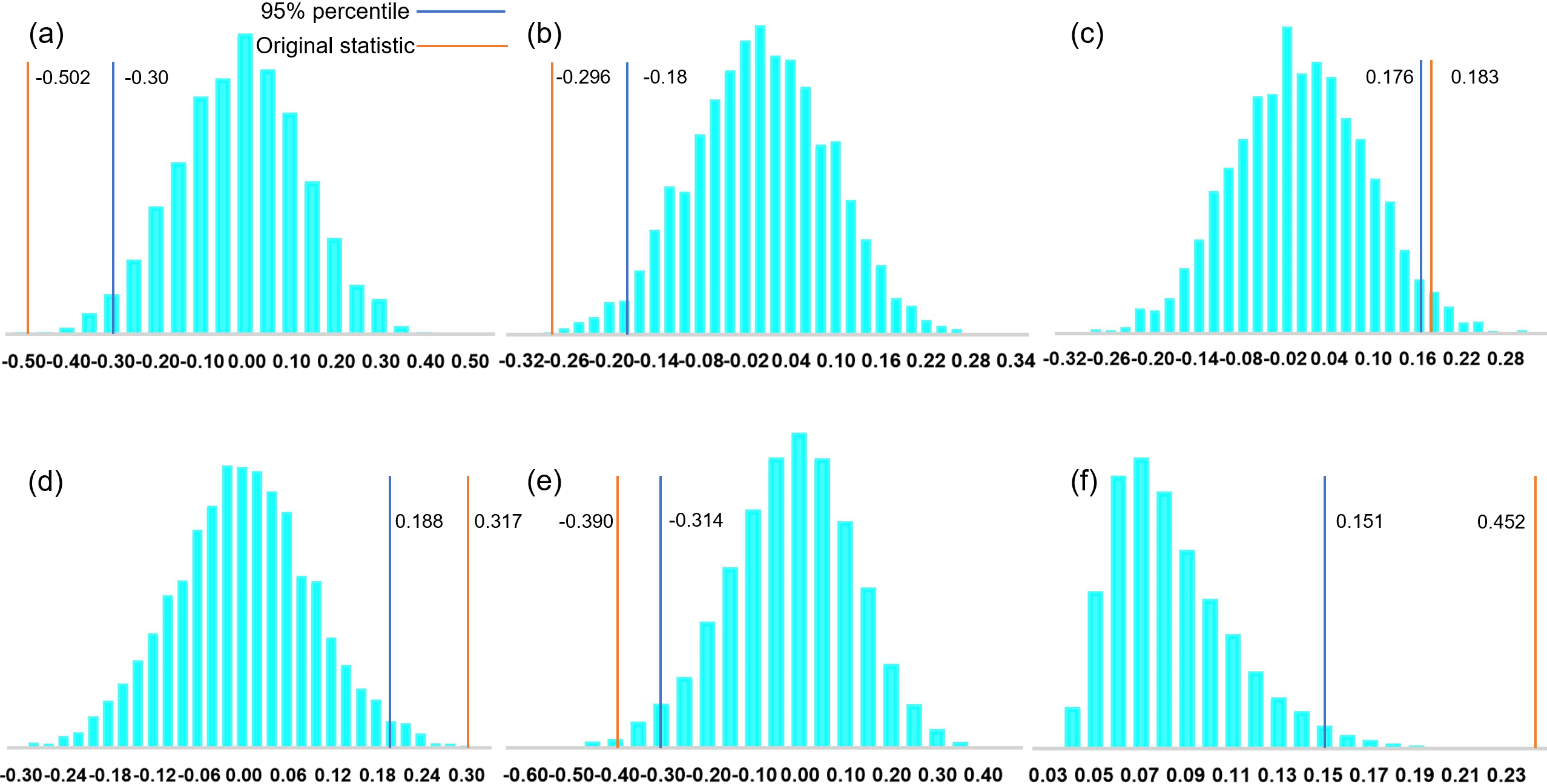


Sup Fig. 5. Null distribution generated by permutation test. (a, b, c, d, e) The distribution of Spearman correlation coefficients (*r* values) between the expression levels of neurotransmitters (a: 5HT1b, b:5HT2a, c:D2, d:DAT, e:mGluR5) and the *t*-map is obtained through permutation testing. (f) The distribution of Spearman correlation coefficients (*r* values) between the percent of variance explained in the response variable by component1 (values of original data: 0.452) and the *t*-map is obtained through permutation testing. Blue lines represent the two-sided 95th percentile of the null distribution generated by the permutation test. Orange lines represent the original statistical measures.


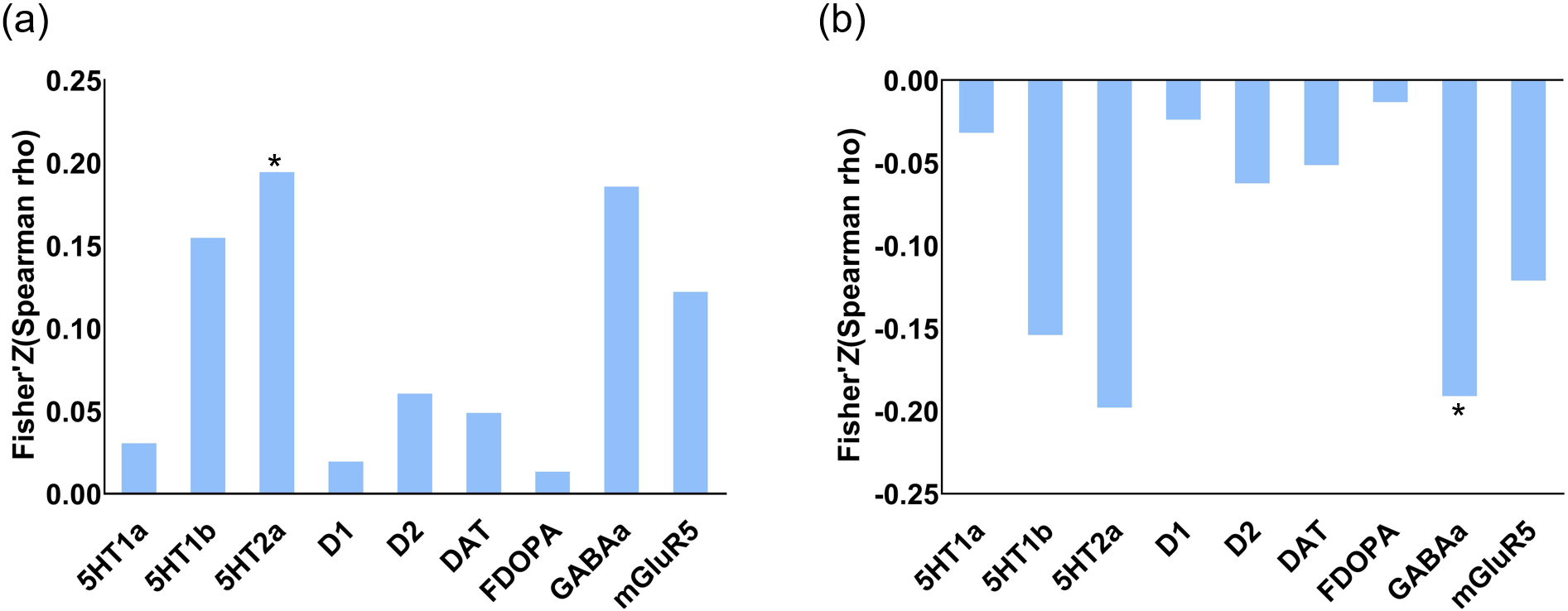


Sup Fig. 6. Association between multi-map and neurotransmitter receptors/transporters. (a) multiplying the SVR feature map with the t-map showing significant differences in cortical thickness, where the values of the t-map remained as the corresponding t-statistic values, and (b) multiplying the SVR feature map with a binary version of the t-map, where 1 indicates significant cortical thickness differences in the respective brain regions.

**Sup results 5 Brain age prediction model trained by all HCs and model performance**

The correlation between chronological age and predicted age are presented in Sup Fig. 7a and Sup Fig. 7b. The difference of brain-PAD between healthy controls and MDD patients is significant (*t*=2.29, *p*=0.0223, Cohen’s d=0.1856, 95% CI: 0.03-0.34, Sup Fig. 7d). And permutation test for difference of brain-PAD is significant (*p*=0.0017). Compare with healthy controls, MDD patients shows significant higher predicted age (*p*=0.0219, Cohen’s d=0.1862, 95% CI: 0.03-0.35), while there is no difference in chronological age (*p*=0.2644, Sup Fig. 7c).


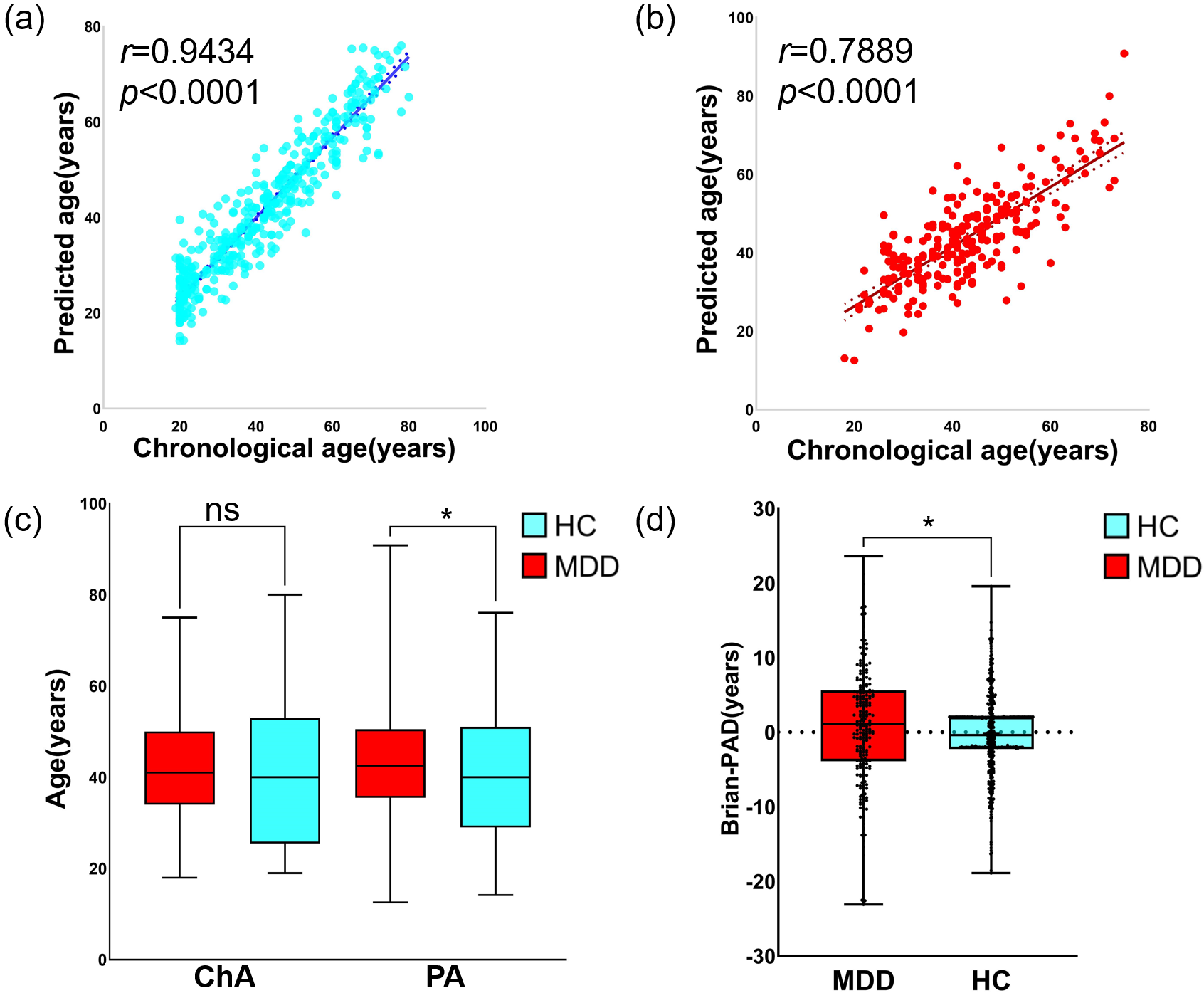


Sup Fig. 7. Performance of brain age prediction. (a) Positive correlation between the chronological age and predicted age in healthy controls. (b) Positive correlation between the chronological age and predicted age in MDD patients. (c) Group comparison between MDD patients and healthy controls and there is no significant difference in chronological age between two groups, while the predicted age in MDD patients is significantly higher than that in healthy controls. (d) Group comparison of the brain-PAD between MDD patients and healthy controls and brain-PAD in MDD patients is significantly higher than that in healthy controls. Ns means that the difference between MDD and HC is not significant. *, *p*<0.05.
